# Optical Waveguiding Charge-Transfer Cocrystals: Examining the Impact of Molecular Rotations on Their Photoluminescence

**DOI:** 10.1021/jacs.4c15957

**Published:** 2025-02-26

**Authors:** Armando Navarro-Huerta, Takumi Matsuo, Alexander S. Mikherdov, Jan Blahut, Erika Bartůňková, Pingyu Jiang, Martin Dračínský, Simon Teat, Mingoo Jin, Shotaro Hayashi, Braulio Rodríguez-Molina

**Affiliations:** a Instituto de Química, Universidad Nacional Autónoma de México, Coyoacán, Mexico City 04510, Mexico; b School of Engineering Science, Kochi University of Technology, 185 Yosayamada Miyanokuchi, Kami, Kochi 782-8502, Japan; c Institute for Chemical Reaction Design and Discovery (WPI-ICReDD), Hokkaido University, Sapporo, Hokkaido 060-8628, Japan; d Institute of Organic Chemistry and Biochemistry, Czech Academy of Sciences, Prague 160 00, Czech Republic; e Advanced Light Source, Lawrence Berkeley National Laboratory, Berkeley, California 94720-8229, United States; f FOREST Center, Research Institute, Kochi University of Technology, 185 Yosayamada, Miyanokuchi, Kami, Kochi 782-8502, Japan

## Abstract

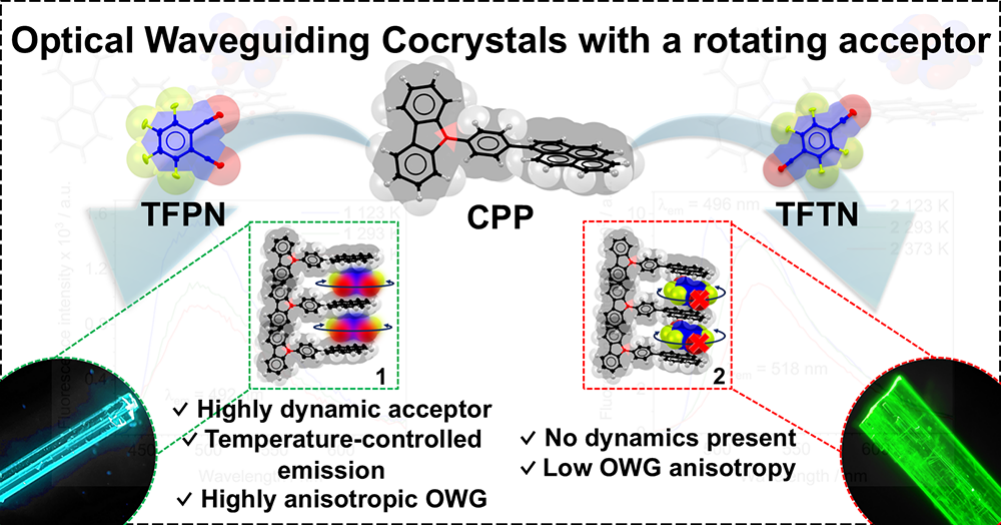

Here, we present the first example of a binary optical waveguiding (OWG) cocrystal with large anisotropy featuring a fluorinated acceptor molecule (CPP-TFPN, **1**) with on-plane rotational dynamics, confirmed by solid-state NMR (^19^F *T*_1_) and theoretical calculations. Spatially resolved microphotoluminescence and variable-temperature photoluminescence experiments allowed us to examine the OWG performance and photophysical properties of both single crystals and bulk microcrystalline samples. A comparison with an analogous cocrystal containing a regioisomeric acceptor (CPP-TFTN, **2**) revealed that the photoluminescence characteristics of **1** are associated with the rotational motions of the acceptor, offering insights into how the molecular motion changes this property.

## Introduction

The increasing demand for high-performance materials in optoelectronic devices has led to significant interest in organic cocrystals due to their ease of processing and lightweight nature. Unlike traditional crystalline materials, multicomponent systems provide customized morphological^1−3^ and photophysical^4,5^ properties by altering molecular combinations and stoichiometries.^6^ This approach takes advantage of the crystal engineering precepts through the complementary noncovalent interactions,^7^ ultimately leading to tunable emissive materials either by changing the initial materials^8^ or by fusing different crystalline materials following a lattice-mismatch heteroepitaxial strategy.^9,10^

Notably, π-stacking is a crucial noncovalent interaction (NCI) implemented in multicomponent conjugated systems that plays a significant role in modulating the resulting photophysical properties.^11^ The pairing of electron donors (D) and acceptors (A) often leads to solids exhibiting a red-shifted photoluminescence (PL) emission;^12^ however, this combination can compromise the resulting PL intensity due to the facilitation of nonradiative pathways.^13,14^ Contrastingly, when a highly luminescent donor is combined with a mild electron-withdrawing fragment with a low tendency to form paramagnetic species, the PL is typically preserved.

The propagation of NCIs along specific crystallographic axes might result in anisotropic fluorescent single crystals capable of transmitting light in a specific direction, known as optical waveguides (OWGs),^15^ with potential application in nanophotonics^16,17^ and data transmission.^11^ Remarkable works of organic OWG materials based on cocrystals and molecular organic crystals have been reported using the directionality of the halogen^18−20^ and hydrogen^21−23^ bonds to propagate NCIs from the crystal packing both as one-^24^ or two-dimensional^25^ single crystals. However, the π-stacking provides a platform for the embedding of molecular fragments with different degrees of electron-accepting characteristics.^26^

Our approach to fine-tuning and enhancing the photophysical properties of crystalline materials relies on the incorporation of molecular rotational motions within the structure via π-stacking of D/A pairs. It has been postulated that internal motions contribute to lattice relaxation via Brownian motion. For example, a recent study highlights that the molecular motion of small, symmetric fragments, like the 1,4-tetrafluorobenzoquinone component in a cocrystal, serves as a sink for radiative relaxation pathways, improving photothermal conversion properties.^27^

Tetrafluorophthalonitrile (TFPN) and tetrafluoroterephthalonitrile (TFTN) are small molecular building blocks previously used^3,10^ to assemble photoluminescent CT cocrystals due to their low degree of charge transfer character. Previously, it was reported that analogue tetrachlorophthalonitrile can display order–disorder phase transitions in CT cocrystals, which could be taken as the first approximation that other building blocks with smaller halogen atoms may show enhanced molecular motions, which could be evidenced by ^19^F solid-state NMR.^28^

## Results and Discussion

### Structural Analysis and Crystal Morphology

**Figure 1:**
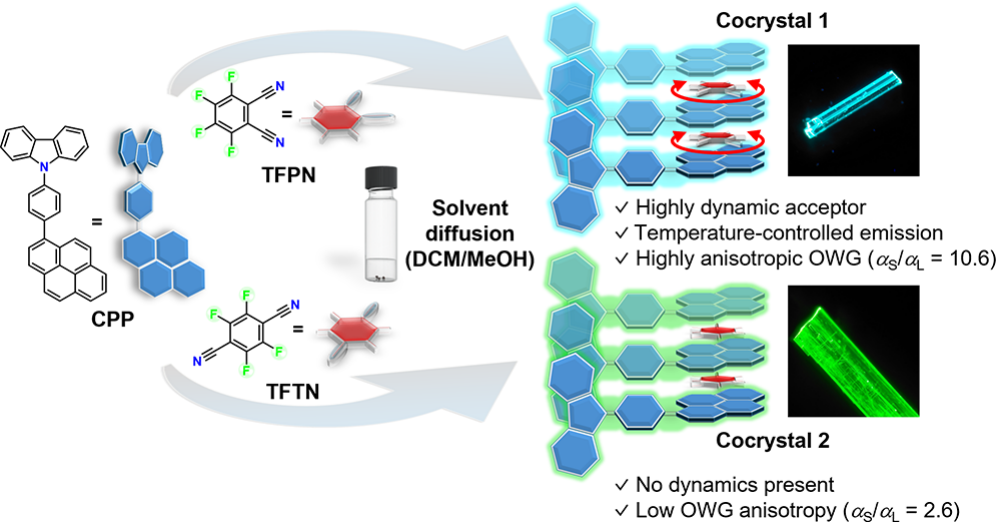
Synthesis of cocrystals with fluorinated acceptors to obtain organic optical waveguiding materials featuring tunable emission using a temperature stimulus via in-plane rotations of a small acceptor molecule.

**Figure 2:**
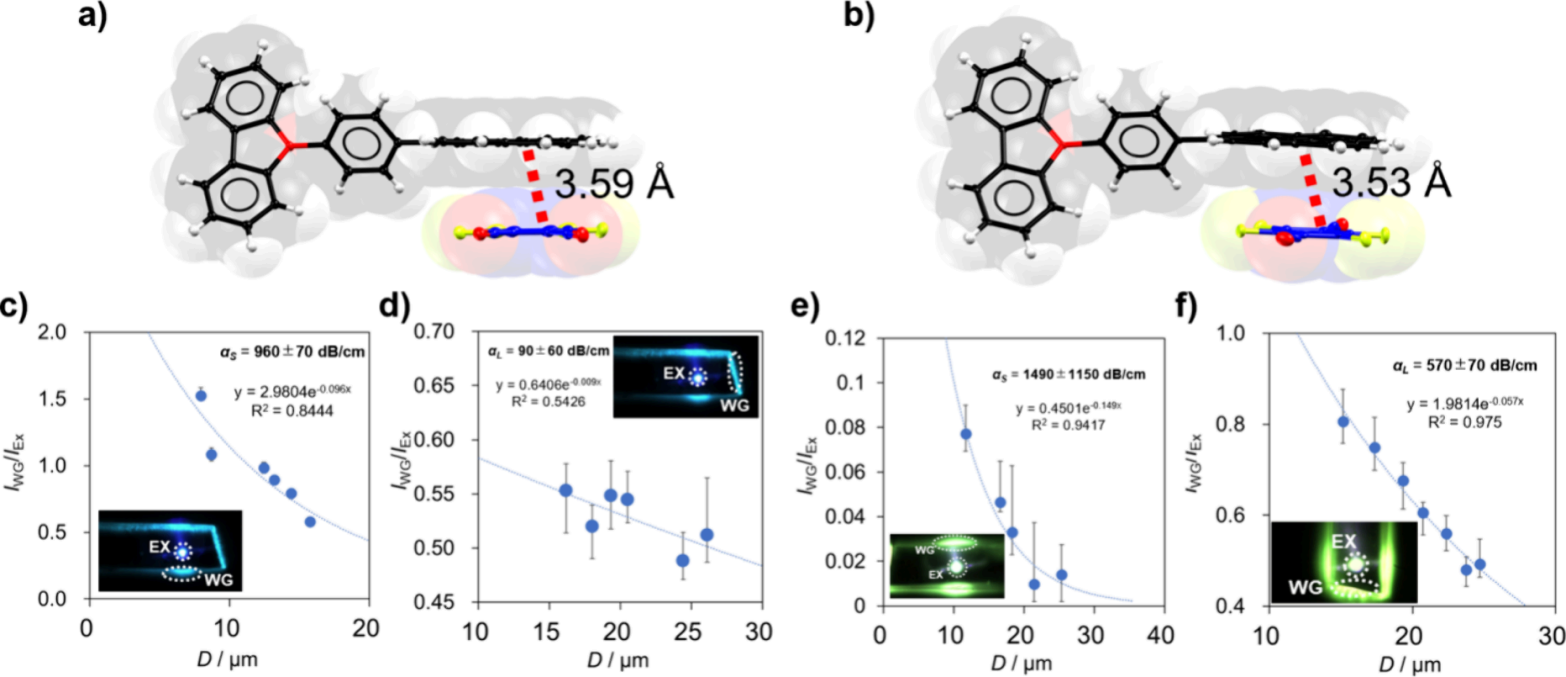
π-Stacking and D/A distances in (a) cocrystal **1** and (b) cocrystal **2**. Ratios of emission intensity between excitation and waveguided positions in the long and short axes plotted as a function of the waveguided distance *D* for (c, d) cocrystal **1** and (e, f) cocrystal **2**. Pictures of the measurements by the OWG in the crystals are indicated as insets in each plot (λ_monitored_ = 500 nm).

In line with this reasoning, we present the characterization of two organic cocrystals: **CPP-TFPN** (**1**) and **CPP-TFTN** (**2**). As depicted in Figure 1, both were grown with the same fluorescent, polycyclic donor (CPP, after carbazole-phenylene-pyrene) through a liquid–liquid diffusion method (details of the synthesis of compound and crystallization are in the Supporting Information), yielding acicular single crystals. Under 254 nm UV light, cocrystal **1** produced turquoise-blue, fluorescent crystals, while cocrystal **2** displayed green fluorescence (Figure S5). Structural characterization was performed using variable-temperature single-crystal X-ray diffraction (VT SCXRD) using synchrotron radiation at 100, 200, and 300 K (Tables S1 and S2), revealing a 1:1 D/A stoichiometry in both cases. The interplanar distances between the pyrene core and the acceptors were 3.59 Å for **1** and 3.53 Å for **2** (Figure 2a,b).

The morphology of the crystals was further studied using the growth morphology method in Materials Studio. The calculated attachment energies (*E*_att_; Table S3, Figure S6) show that crystal growth occurs along the [100] crystallographic direction, consistent with the macroscopic shape of the single crystals. Additionally, energy framework calculations using CrystalExplorer 21.0,^29^ featured in Figure S7, indicated similar D/A interaction energies in both solids, approximately −52 kJ/mol for **1** and −54 kJ/mol for **2**.

### Characterization of the Optical-Waveguiding Performance

Interestingly, under UV irradiation, the single crystals of both solids exhibited pronounced brightness at the tips, a characteristic feature of the OWG materials. To evaluate their performance, space-resolved microphotoluminescence (μPL) measurements were conducted at room temperature (details in the Supporting Information). To determine the facet for crystal irradiation, the transition dipole moments (TDMs) of the emissive CPP molecules were calculated in both systems; these vectorial quantities determine the probability of an electronic transition along a specific direction. The orientation of the resulting vectors, shown in Figure S8, is perpendicular to the π-stacking propagation between the donor and the fluorinated acceptors. Previous studies have shown that when the electric field vector of the incident light and the TDM of the chromophore are perpendicular, light reabsorption is maximized, improving waveguiding performance.^30^

Photoirradiation experiments were conducted along both the long and short crystal axes with varying distances (*D*) between the excitation and waveguided positions, as depicted in the input images of Figure 2. Additional fluorescence images (Figure S9) display these distances. The recorded μPL spectra for both axes at the excitation and waveguiding positions, along with the crystal orientation, are shown in Figures S10 and S11.

The intensity ratios between waveguiding and excitation positions (*I*_WG_/*I*_EX_) were plotted (Figure 2c–f) and fitted using the relationship *I*_WG_/*I*_EX_ = *A* exp(−α*D*) to calculate the optical loss coefficients (α). For **1**, the loss coefficients are 960 ± 70 and 90 ± 60 dB cm^–1^ for the short (α_S_) and long axis (α_L_), respectively. For **2**, the loss coefficients are α_S_ = 1490 ± 1150 dB cm^–1^ and α_L_ = 570 ± 70 dB cm^–1^. These values lie in the order of magnitude of previously reported OWG materials based on pyrene and carbazole.^20,30−32^

The ratio of the loss coefficients (α_S_/α_L_) quantifies the degree of anisotropy in an OWG crystal.^18^ Based on the determined values, **1** exhibits an anisotropic waveguiding ability of 10.6, whereas for **2**, this parameter is 2.6. Recent reports^22,30,33,34^ on OWG crystalline materials imply that values α_S_/α_L_ > 3.9 describe a highly anisotropic material with potential applications for highly regulated organic optical gates. The higher anisotropy in **1** (4-fold from **2**) may be attributed to slightly different directions of the TDM with respect to the incident electric field vector (Figure S8). Figure S12 displays overlapping of the absorption and emission spectra for both cocrystals. Cocrystal **1** portrays a 31% higher reabsorption than cocrystal **2**, which could be a feasible reason for the higher anisotropy of the OWG. Additional measurements of the α_L_ for cocrystal **2** (Figure S13) were performed using different laser wavelengths; nevertheless, the optical loss coefficient decreased, causing a change in the performance.

### Variable-Temperature Photoluminescence Experiments in Polycrystalline Samples

**Figure 3:**
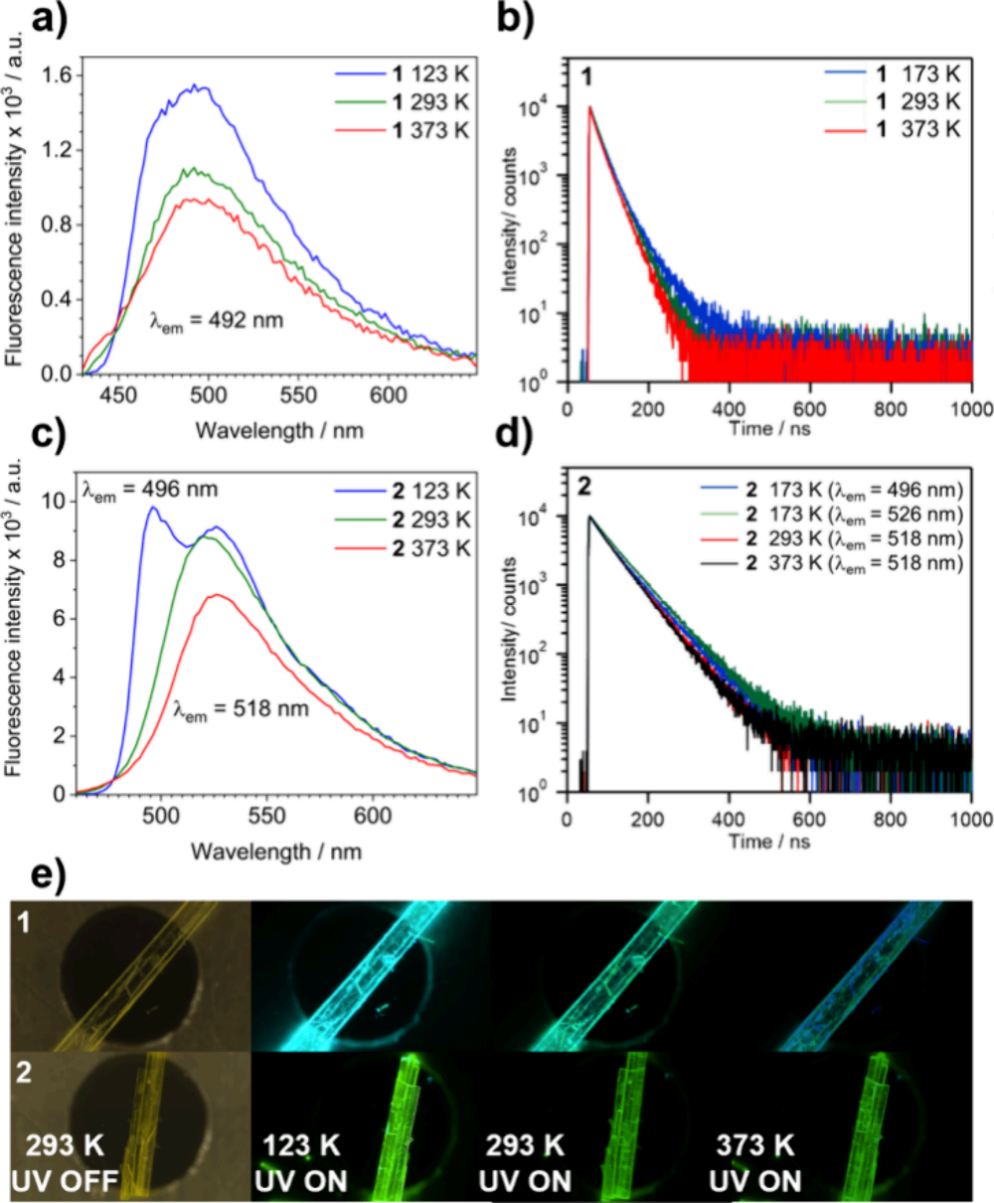
Variable-temperature comparative PL spectra of (a) cocrystal **1** and (c) **2** indicating the respective emission wavelength for the maxima. The time-resolved PL decay profiles of the cocrystals are depicted in panels b for **1** and d for **2** using a 375 nm pulse laser. (e) Single-crystal fluorescence micrographs at variable temperatures of cocrystals, highlighting the changes in PL emission.

Since μPL measurements on single crystals cannot be performed at low temperatures due to technical limitations, we investigated further the emission of these cocrystals by variable-temperature PL experiments on bulk polycrystalline samples. First, the crystalline phase of the solids was confirmed through powder X-ray diffraction (PXRD) (Figure S14). The spectra of **1** (Figure 3a) confirmed the persistence of the emission maximum at 492 nm (λ_ex_ = 372 nm, CIE 0.064, 0.217, Figure S15) across the temperature range. The increasing PL intensity followed a trend inversely proportional to the temperature, as illustrated in Figure S16a, showing a linear 65% increase. In contrast, a new PL maximum emerged for **2** at 123 K (Figure 3c) with a wavelength of 496 nm (λ_ex_ = 397 nm, CIE 0.059, 0.829, Figure S15) compared to the 518 nm maximum observed at 293 and 373 K. The new maximum that is evident for **2** and barely noticeable for **1** may result from restricted molecular motions or freezing conformations at low temperatures, which enable new excited states, as suggested by Crespo-Otero et al.^35−37^ The PL intensity of **2** varied by 44% between the highest and lowest temperatures (Figure S16b), following a nonlinear trend, indicating a different PL relaxation process in this solid. Moreover, the PL quantum yields of the solids at room temperature were 23% (λ_ex_ = 472 nm) for cocrystal **1** and 26% (λ_ex_ = 496 nm) for cocrystal **2**.

Time-resolved decay measurements were performed to further investigate the PL of the solids. Figure 3b,d shows the decay curves for both cocrystals at 173, 293, and 373 K. The tail-fitting model revealed an average fluorescent lifetime (τ_avg_) between 27 and 32 ns for **1**, while **2** showed significantly longer lifetimes between 62 and 69 ns. The complete fitting parameters are provided in Table S4. Single-crystal fluorescence micrographs were collected by using a LINKAM cryosystem to visualize these changes, confirming the PL intensity variations over the temperature range. These pictures are featured in Figure 3e.

### Characterization of the Charge-Transfer Phenomena

**Figure 4:**
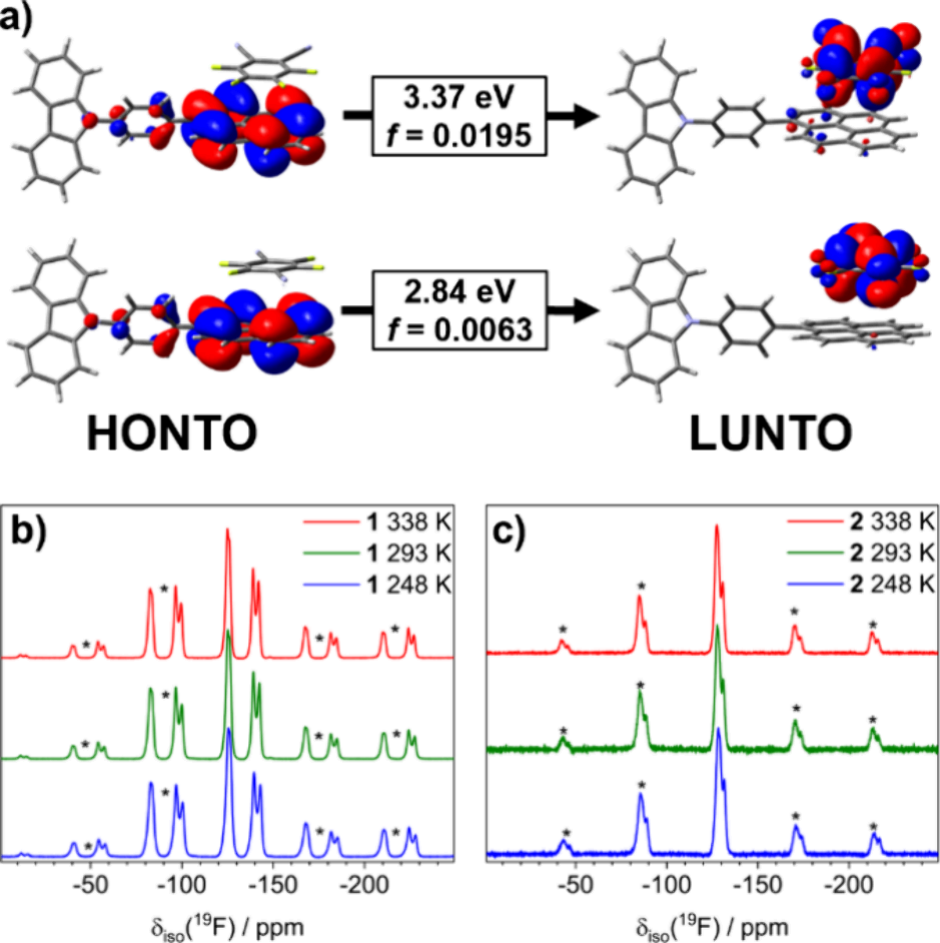
(a) Frontier natural transition orbitals for dyads of the cocrystals (up: **1**; down: **2**) at the M06-2X/6-311(d,p) level of theory with the energy associated with the S_0_ → S_1_ transition and oscillator strength (f). VT ssNMR ^19^F MAS spectra of cocrystals (b) **1** and (c) **2**. The acquisition parameters are indicated in the Supporting Information. The asterisk indicates the spinning sidebands of the central set of signals (20 kHz).

Absorption spectroscopy using diffuse reflectance, highlighted in Figure S17a, revealed two broad absorptions, especially for cocrystal **2**. From these, the electronic optical gaps (*E*_g_'s) were calculated using Tauc plots, yielding 2.93 eV for **1** and 2.47 eV for **2** (Figure S17b). These results suggest an intermolecular charge-transfer (ICT) effect between the D/A dyads, which is enhanced in cocrystal **2** due to the molecular symmetry of the acceptor,^3,4^ facilitating the electronic transitions from the donor to the acceptor. To confirm this, the ground-state geometries of the D/A dyads were optimized using density functional theory (DFT) and the Perdew–Burke–Erzenhof (PBE) functional.^38^ The excited states and electronic transitions were then calculated using time-dependent (TD) DFT at the M06-2X^39^/6-311G(d,p) level of theory in Gaussian09.^40^ The results showed that the S_0_ → S_1_ transition corresponds to an intermolecular charge-transfer state, with energies of 3.37 eV for **1** and 2.84 eV for **2**. A further calculation (Figure S31) with the dimer of cocrystal **1** and the TFPN rotated by 15° indicates that the change in the PL spectra can be attributed to slight changes in the conformations of the D/A components in the cocrystal. The natural transition orbitals (NTOs) involved are in the CPP and acceptor moieties, as depicted in Figure 4a. The NTOs of the starting materials were calculated at the same level of theory and are shown in Figure S18, pointing out a significant reduction in the energetic gap of the cocrystals compared to the starting materials.

Electron spin-resonance (ESR) spectroscopy in the solid state ruled out the presence of long-lived paramagnetic species even at temperatures as low as 77 K, as shown in Figure S19. It has been reported that the relaxation times of radical species can be highly affected due to the presence of molecular motions,^41^ as shall be described further in this work. Moreover, the degree of CT (ρ), a measurement of the ionicity of crystalline materials, was calculated through measurements of bond distances in SC XRD structures,^5^ revealing values of 0.035e and 0.100e for cocrystals **1** and **2**, respectively. The results are condensed in Table S5 and are consistent with our experimental evidence through UV–vis spectroscopy. Additionally, the Fourier-transformed infrared (FTIR) spectra of the cocrystals further confirmed the low degree of ICT, inferred from the minimal shifts in the C≡N stretching bands of the cyano groups compared to the starting materials, consistent with previous reports (Figures S20 and S21).^42,43^

The changes in PL intensity across the temperature range prompted us to explore the feasibility of nonradiative pathways, specifically through molecular motions of small, symmetric fragments. A precedent by Garcia-Garibay et al. demonstrated that thermally driven molecular motions of a rotating fragment can detrimentally affect PL intensity in crystalline solids.^44^

### Assessment of the Molecular Motion through Solid-State NMR

To assess molecular motions in cocrystals **1** and **2**, we examined the variable-temperature single-crystal X-ray diffraction and NMR data for both solids. First, the thermal stability of the cocrystals was studied through differential scanning calorimetry coupled with thermogravimetric analysis (DSC/TGA), depicted in Figure S22. Then, solid-state nuclear magnetic resonance (ssNMR) spectroscopy was carried out. The resulting ^19^F MAS (magic-angle spinning) spectra (Figure 4b,c) at variable temperatures revealed no significant differences in the pattern and chemical shifts of the fluorinated acceptors. The three main sets of signals for **1** appeared at −125.6, −139.5, and −143.0 ppm, while for **2,** they were at −128.3 and −131.5 ppm. Additionally, the cross-polarization (CP MAS) pulse sequence on ^13^C nuclei shows a series of aromatic signals (δ(^13^C) > 100 ppm) in both solids, as featured in Figure S23.

**Figure 5:**
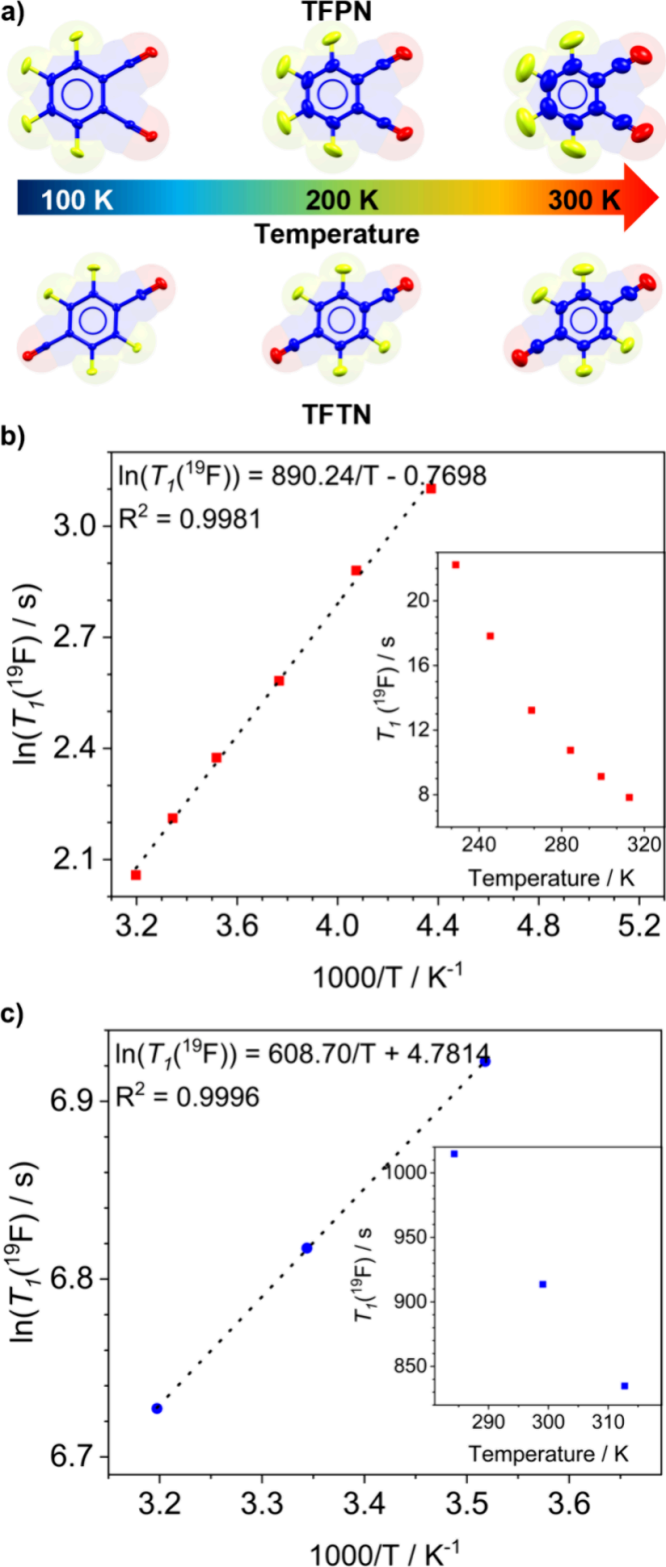
(a) Comparison of the elongation of the thermal ellipsoids in the TFPN and TFTN acceptor fragments in cocrystals **1** and **2**, respectively, along the temperature interval. Thermal ellipsoids were drawn at the 50% level probability. Arrhenius plots using ^19^F *T*_1_ relaxation times through ssNMR saturation-recovery experiments in (b) cocrystal **1** and (c) cocrystal **2**. As insets are depicted, the *T*_1_ times vary as a function of the temperature for each graph.

Regarding the SC XRD data, we observed that increasing the temperature leads to a more pronounced elongation and size variation in the thermal ellipsoids of the acceptor molecules in **1** compared to its TFTN analog. The deformation of the ellipsoids around the fluorine atoms occurs tangentially to the acceptor molecule, as shown in Figure 5a.

### ^19^F *T*_1_ Relaxation Measurements and Calculated Energy Barriers

As previously reported by Beckmann et al.^45,46^ and us,^27^ molecular localized motions of fluorinated fragments can be studied through saturation-recovery experiments, which determine the ^19^F *T*_1_ values. Using the Arrhenius equation to plot relaxation times as a function of temperature, ln(*T*_1_) = *E*_a_/*RT* + ln(*A)*, we constructed the graphs in Figure 5b,c. Due to the high relaxation times of the TFTN cocrystal, only three *T*_1_ measurements were collected. In contrast, the relaxation behavior of **1** did not display the minimum associated with the concordance between rotational motion frequencies and the Larmor frequency of the spectrometer. Further measures would require a broader temperature range, which is currently beyond the capacity of the employed spectrometer. Also, the behaviors of the *T*_1_ times as a function of the temperature are summarized in Table S6 and depicted as insets, showing distinct behaviors in the two cocrystals, with the relaxation times for **1** being up to 2 orders of magnitude shorter than those in **2**. The differences in the *T_1_* relaxation rates between cocrystals indicate distinct molecular dynamics due to different molecular microenvironments surrounding the fluorine atoms.

The rotational energy profiles of the two acceptors were also determined by using optimized geometries of molecular clusters through DFT calculations with the B3LYP functional. The resulting profiles are shown in Figures S24 and S25. For the TFPN in cocrystal **1**, the geometry optimizations provided two local minima corresponding to +60 and −60° rotation from the global minimum at 0° and relatively low rotational barriers of 5.9 and 7.4 kcal/mol. In contrast, for cocrystal **2**, only one minimum at −60° was found with a rotational barrier of 17.5 kcal/mol. These calculations support the experimental data indicating that the rotation of TFPN in cocrystal **1** is allowed and that the noncovalent interactions and steric hindrance in **2** (Figures S26 and S27) significantly hinder the in-plane rotation of TFTN.

Finally, to assess the possible contributions of the phenylene rings in the CPP core to overall dynamics, we performed ssNMR experiments on phenylene-deuterated cocrystals **1-***d*_**4**_ and **2-***d*_**4**_ using quadrupolar spin–echo measurements for ^2^H nuclei. The resulting spectra for both cocrystals, shown in Figures S28 and S29, display a typical Pake pattern, indicating the absence of molecular rotations within the time scale for this nucleus (10^4^–10^7^ Hz).^47^ Additionally, an analysis of the NCI around the phenylene revealed significant steric hindrance with the surrounding CPP molecules, as shown in Figure S30.

## Conclusions

This work provides a comprehensive analysis of two new CT cocrystals with great OWG properties. Cocrystal **1** exhibits anisotropy up to 4 times higher than that of cocrystal **2** due to the orientation of the transition dipole moment and higher reabsorption favored by its crystal packing. Notably, a significant and linear increase in photoluminescence (PL) at 123 K in cocrystal **1** can be attributed to the reduction of the in-plane rotational motion of its TFPN component at low temperatures, as supported by extensive solid-state NMR relaxation measurements and DFT calculations. These findings set a precedent for cocrystals with excellent OWG and thermally driven photoluminescent properties, suitable for applications such as optically regulated gates.

## Supporting Information Available

Synthetic and experimental procedures; ^1^H and ^13^C NMR spectra of compounds; microscope photographs; thermal analyses; theoretical calculations; ssNMR ^13^C CP MAS, VT ^19^F MAS, and VT ^2^H NMR spectra; ^19^F *T*_1_ relaxation times; final crystallographic refinement parameters; PXRD diffractograms; Tauc plots; PL model fittings; VT solid-state ESR; and room temperature FTIR spectra (PDF)
