# Supplementary material for: Optical Waveguiding Charge-Transfer Cocrystals: Examining the Impact of Molecular Rotations on Their Photoluminescence
Source: J Am Chem Soc. 2025 Feb 26;147(10):8343–9. doi: 10.1021/jacs.4c15957 (PMC11912476; doi:10.1021/jacs.4c15957)
Supplement: Supplementary file 1 — ja4c15957_si_001.pdf [file ja4c15957_si_001.pdf]

Supporting Information

Optical Waveguiding Charge-Transfer Cocrystals: Examining the Impact of Molecular Rotations on Their Photoluminescence

Armando Navarro-Huerta[a], Takumi Matsuo[b], Alexander, S. Mikherdov[c], Jan Blahut[d], Erika Bartůňková[d], Pingyu Jiang[c], Martin Dračínský[d], Simon Teat[e], Mingoo Jin[c*], Shotaro Hayashi,[b,f*] Braulio Rodríguez-Molina[a*]

[a] *Instituto de Química, Universidad Nacional Autónoma de México, Coyoacán 04510, Mexico City, Mexico.*

[b] *School of Engineering Science, Kochi University of Technology, 185 Yosayamada Miyanokuchi, Kami, Kochi, 782-8502, Japan.*

[c] *Institute for Chemical Reaction Design and Discovery (WPI-ICReDD), Hokkaido University, Sapporo, Hokkaido, 060-8628, Japan.*

[d] *Institute of Organic Chemistry and Biochemistry, Czech Academy of Sciences, 160 00 Prague, Czech Republic.*

[e] *Advanced Light Source, Lawrence Berkeley National Laboratory, Berkeley, California 94720-8229, United States*

[f] *FOREST Center, Research Institute, Kochi University of Technology, 185 Yosayamada, Miyanokuchi, Kami, Kochi, 782-8502, Japan*

*brodriguez@iquimica.unam.mx

*mingoo@icredd.hokudai.ac.jp

*hayashi.shotaro@kochi-tech.ac.jp

Materials and methods

Reagents and solvents were purchased from Sigma-Aldrich®. TFPN and TFTN were employed as received. Synthesis of 9-(4-iodophenyl)-*9H*-carbazole and 9-(4-iodophenyl)-*9H*-carbazole-d_4 was carried out as previously reported by our group.[S1] NMR signals and FTIR spectra are consistent with the data given in the reference. Reactions were monitored through TLC using silica gel plates 60 F_{254} purchased from Merck®. Spots were detected by UV-light absorption. Reactions were carried out under an inert atmosphere using nitrogen (N_2). Solution ^{1}H and ^{13}C experiments were recorded at room temperature using a Bruker AVANCE III 400 or Jeol Eclipse 300. The spectroscopic data is referenced to $CDCl_3$ (^{1}H: δ = 7.26 ppm, s; ^{13}C: δ = 77.0 ppm), otherwise noted. High-Resolution Mass Spectrometry was obtained in a Jeol JMS-AccuTOF JMS-T100LC spectrometer, ionization mode: Direct Analysis in Real Time (DART). FTIR spectra experiments were recorded in a Bruker Tensor 27 in ATR mode, equipped with a diamond tip in the spectral window from 4000 to 500 cm^{-1}. Uncorrected melting points were determined in a Fisher-Johns melting point apparatus, otherwise noted.

Synthesis of cocrystals

Synthesis of cocrystal 1

In a 20 mL vial, 20 mg of pure **CPP** (0.045 mmol, 1 eq.) and 27 mg of **TFPN** (0.135 mmol, 3 eq.) were placed. Then, 8 mL of dichloromethane were poured into the vial. The vial was sealed and heated to 60 °C to ensure full solubility of solids. The vial was then allowed to cool to room temperature; afterwards, 8 mL of methanol were very slowly and carefully poured from the walls, forming a layer of MeOH on top of the DCM. Finally, the vial was half-sealed to allow slow evaporation of the solvents. Small, acicular, yellow crystals grew over time (ca. 4 days). Yield: 27.7 mg. Melting point (DSC): 195 °C (From 150 °C the expelling of **TFPN** from the crystalline network occurred).

Synthesis of cocrystal 2

The same procedure used for **1** is employed to afford crystals of **2**. The same quantities (20 mg of **CPP** and 27 mg of **TFTN**) were placed in the 20 mL vial. After ca. 4 days, acicular, green crystals were formed, which were also vacuum filtered in a Hirsh funnel. Yield: 24 mg. Melting point (DSC): 160 °C (Thermal stability of this compound is held up to 125 °C).

Synthesis of cocrystals with CPP*d*$_4$

For the synthesis of the deuterated cocrystals (using **CPP*d*$_4$**), the same quantities of each reagent were employed as described in the procedures. No noticeable physical changes were found for the cocrystals with deuterated and natural abundance **CPP**.

Thermogravimetric analysis and differential scanning calorimetry

Coupled DSC and TGA measurements were carried out in a Netzsch STA 449 F2 Jupiter thermal analyzer under nitrogen atmosphere. A heating ramp comprising from 25 to 300 °C was employed with a heating rate of 10 °C/min. The samples were loaded in 5 mm-diameter aluminum crucibles with a hole in the cap.

Variable temperature photoluminescence experiments

Photoluminescence (PL) emission and excitation spectra were recorded using an Edinburgh Instruments FLS1000 Photoluminescence Spectrometer. For PL emission and excitation, a Xe2 Xenon Arc Lamp was utilized. Emission decay profiles were obtained employing the μF2 Microsecond Flashlamp, maintaining consistent excitation/emission bandwidth parameters as for emission and excitation measurements.

Room temperature (RT) measurements were conducted on crushed crystals utilizing the N-J03 Front-Face sample holder for solid samples. In these measurements, a 0.5 nm excitation/emission bandwidth and 0.1 dwell time were used, with data accumulation carried out 5 times. Emission intensity was collected up to 10000 counts for RT emission decay measurements.

Variable temperature (VT) measurements on single crystal particles were performed using the Olympus BX51WI microscope linked to the FLS1000 Spectrometer via light fibers, accompanied by the Olympus DP74 digital camera and LINKAM cryosystem. In VT measurements, a 3.5 nm excitation/emission bandwidth and 0.2 dwell time were used, with data accumulation also repeated 5 or 10 times. Emission intensity was collected up to 2000 counts for VT emission decay measurements.

Space-Resolved micro-Photoluminescence of single crystals

Space-resolved μ-PL spectra were measured by an analyzing system [405 nm UV laser OptoSigma LDU33-405-3.5 (Sigma-Koki), micro-PL spectra using a USB400 and a R200-

7-UV-VIS probe] recorded by a CCD (detector: Sony ILX511B linear silicon CCD array). The excitation laser was filtered with a band-pass filter (YIF-BA460IFS) and focused on the crystal samples with an objective (×50, NA = 0.80 or ×20, NA = 0.45). The crystal samples were excited at 5.0 μW. The collected PL was then guided to a spectrometer (Lambda Vision SA-100 A) and recorded by a CCD (Detector: Hamamatsu photonics S11151-2048 CCD linear image sensor). The system is illustrated below

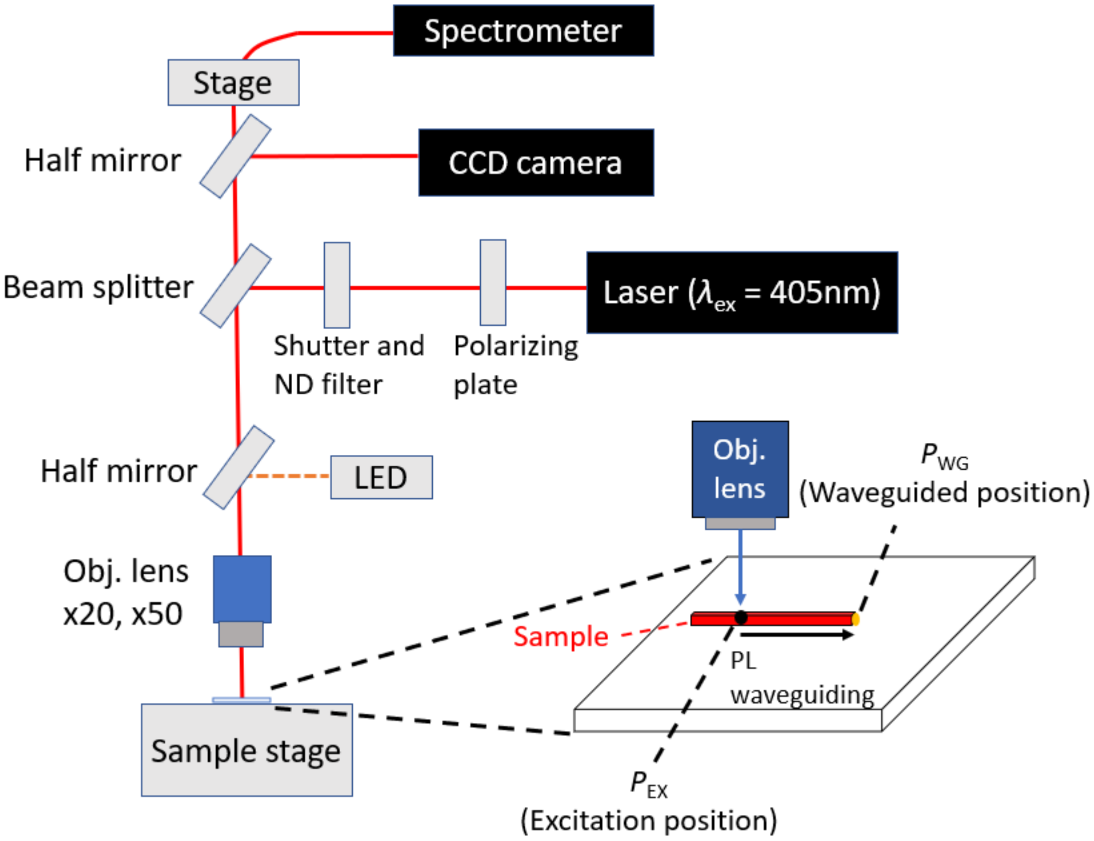

Moreover, the ratio between the intensity at the excitation point (I_{EX}) and waveguided position (I_{WG}) was plotted as a function of the distance between both points (D). The ratios were adjusted to Equation S1

$$I_{WG}/I_{EX} = A\exp(-\alpha D) \qquad \text{Eq. S1}$$

Where A is a constant and α is the loss coefficient, which provides information about the performance of the solid. For **1**, the measured distances D were 7.9, 8.7, 12.4, 13.2, 14.4, and 15.7 μm in the short-axis, and 16.2, 18.0, 19.3, 20.5, 24.4, 26.1 μm across the long-axis.

Whilst for **2**, the corresponding distances D were 25.4, 27.9, 29.6, 32.6 and 33.3 μm in the short-axis, and 15.1, 17.4, 19.3, 20.7, 22.4, 23.8 and 24.7 μm in the long-axis.

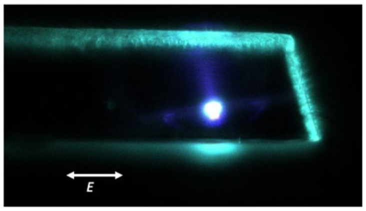

Polarization (E) direction of the excitation laser light. All fluorescent images in the manuscript follow this direction.

Variable Temperature Single Crystal X-Ray Diffraction and Powder X-Ray Diffraction data

X-Ray diffraction data were obtained at variable temperature from a Bruker D8 diffractometer equipped with a PHOTON II CPAD detector with synchrotron radiation (λ=0.7288 Å) on beamline 12.2.1 at Advanced Light Source. Cell refinement was carried out using SAINT V8.38A.[S2] Structure solution, final refinement and data output was carried out using SHELX-2014[S3] through direct methods and SHELXLE-Qt5 as graphical software. Non-hydrogen atoms were refined anisotropically and hydrogen atoms were placed in geometrically calculated positions using a riding model, with isotropic thermal parameters U_{iso}(H) = 1.2U_{eq}(C). Crystal structures were generated with Mercury 2023.3.1.[S4] Powder X-Ray diffraction data were collected at room temperature in a Bruker D8 Advance diffractometer, using CuKα radiation (λ=1.5406 Å) and a Linxeye detector. It was operated at 30 kV and 25 mA with a Bragg-Brentano configuration in a 2θ interval of 5-45°, stepsize 0.03°, steptime 0.06 s.

Solid-state NMR Spectroscopy

Solid-state ^{13}C with CP MAS, ^{19}F MAS and ^{2}H NMR spectra were obtained in a Bruker Avance II with a ^{1}H Larmor frequency of 500 MHz (11.7 T), equipped with a PH MAS DVT 500S1 BL3.2 N-P/F-H probe and Zirconia MAS rotors with VESPEL caps. The ^{13}C spectra were acquired by averaging 5000 transients, with a MAS rate of 20 kHz, delay time of 20 s, pulse width of 4 μs, and a contact time of 2.5 ms at 20 °C. Chemical shifts were referenced to adamantane (δ = 37.7 ppm). ^{19}F MAS spectra were acquired with the average of 64

transients, pulse width 4 μs, MAS rate of 20 kHz and a recycle delay of 5 s, whereas ^{2}H spectra were acquired with a spin-echo pulse sequence, average of 1024 scans with a relaxation delay of 50 s, pulse width of 3 μs and an inversion pulse of 47 μs at the magic angle without spinning of the samples. The ^{19}F T_1 relaxation experiments were acquired on a JEOL 600 MHz spectrometer at 14.1 T field corresponding to the 600 MHz (^{1}H) Larmor frequency, equipped with a 3.2 mm HX probe. The sample was central packed in a 3.2 mm zirconia rotor. Spectra were acquired at an 18 kHz MAS rate using a direct excitation experiment with spin-echo detection. ^{19}F T_1 was obtained using a saturation recovery experiment. The real sample temperature was calibrated using ^{207}Pb shift in solid $Pb(NO_3)_2$ under the same MAS frequency.[S5]

Solid-State Electron-Spin Resonance

Variable-temperature ESR measurements were acquired in a JEOL JES-TE300 spectrometer in X-band, cylindrical cavity TE011, acquisition data program ES-PRITS/TE. At room temperature and 373 K, a quartz tube with an external diameter of 5 mm was used, with microwave power of 4 mW, a center field at 339.39 ± 4.00 mT, a time constant of 0.1 s, a modulation width of 0.79 x 0.10 mT, and a sweep time of 120 s. For measurements at 113 K, a quartz tube with an external diameter of 5 mm was immersed in a coldfinger with N_2, a center field at 320.0 ± 4.0 mT, a time constant of 0.1 s, a modulation width of 0.79 x 0.10 mT, and a sweep time of 120 s. ESR spectra were recorded as the first derivative. To directly compare the spectra intensities of both cocrystals, 0.01 mmol (ca. 6.6 mg) was weighted and placed into the quartz tube at each temperature, with 1 scan acquisition for each spectrum.

Theoretical Calculations

Hirshfeld surface calculations and interaction energies were calculated using *CrystalExplorer 21.5*,[S6] using the *CE*-B3LYP[S7] 6-31G(d,p) functional integrated into the software. Isosurfaces are displayed at 0.002 a.u. The scale factors for electrostatic, dispersion, polarization and repulsion are reported by the authors (k_{elec} = 1.057, k_{disp} = 0.740, k_{pol} = 0.871, k_{rep} = 0.618). SC-XRD structures of **1** and **2** at 300 K were employed for the calculations.

Calculation of the Natural Transition Orbitals (NTOs) was performed using *Gaussian09W*®[S8] Version 9.5, using GaussView[S9] 6.0.16 as graphical visor. Single point

energy calculations were carried out using the SC-XRD molecules of **CPP**, **1**, **2**, **TFTN** (REFCODE: PFTENT10) and **TFPN** (REFCODE: GEYLOL) as starting conformations with optimization of the geometry using the Perdew-Burke-Erzenhof functions. To better consider the effect of NCI, the Minnesota functional M06-2X[S10] was employed, at the level of theory 6-311G(d,p). Generation of NTO images was carried out using the MultiWFN[11] utility integrated in Gaussian at 0.02 au.

Synthetic procedures

General scheme

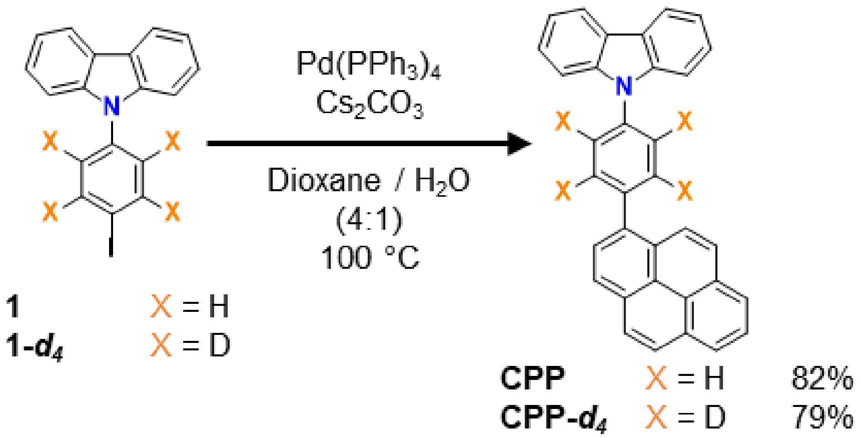

Scheme S1. Synthetic routes to obtain compounds **CPP** and **CPP-*d*₄** reported in this manuscript.

9-(4-(pyren-1-yl)phenyl)-9*H*-carbazole (CPP)

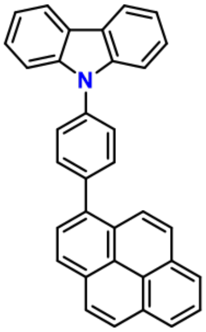

A two-neck round bottom flask was connected to a reflux system with a magnetic stirrer, then it was closed with septa and purged using a pump vacuum. To this system was injected nitrogen gas, the process of purge-pump was repeated one more time. Afterwards, 8 mL of dioxane are added, stirred and bubbled with N_2 for 15 minutes. Then, the next quantities of solid reagents were added: 500 mg (1.35 mmol, 1 eq.) of 9-(4-iodophenyl)carbazole (**1**), 400 mg (1.63 mmol, 1.2 eq.) of pyrene-1-boronic acid, 78 mg (0.07 mmol, 0.05 eq.) of tetrakis(triphenylphosphine)palladium(0), and 882 mg (2.71 mmol, 2.0 eq.) of cesium carbonate. The system was stirred and further bubbled for another 10 minutes with N_2 before heating up to 85 °C. When the system reached 65 °C, 1 mL of deionized H_2O was added. The mixture was left to react for 3 hours, cooled to room temperature and the solvents were evaporated under vacuum. The crude was redissolved with dichloromethane, dried with Na_2SO_4, filtered through celite and supported on silica gel (pore 60 Å, mesh 230-400). An elution gradient was employed to purify the product, starting by

hexanes up to 97:3 hexanes/dichloromethane to obtain an off-white, greenish product, which was further recrystallized from diffusion of a mixture of methanol in chloroform to yield a white crystalline powder and colorless crystals (492 mg, **yield 82%**, **m.p.** 226.6 °C). **^{1}H NMR** (300 MHz, $CDCl_3$) δ: 8.35-8.05 (m, 11H), 7.88 (d, *J* = 8.5 Hz, 2H), 7.78 (d, *J* = 8.5 Hz, 2H), 7.62 (d, *J* = 8.1 Hz, 2H), 7.49 (t, *J* = 7.6 Hz, 2H), 7.34 (t, *J* = 7.6 Hz, 2H). **^{13}C NMR** (75 MHz, $CDCl_3$) δ: 141.0, 140.4, 137.0, 136.8, 132.1, 131.6, 131.1, 131.0, 128.7, 127.9, 127.8, 127.8, 127.6, 127.0, 126.3, 126.2, 125.4, 125.2, 125.1, 124.9, 123.7, 120.5, 120.2, 110.1. **FTIR** (ATR, cm^{-1}) ν: 3044.3, 2924.4, 1922.6, 1676.2, 1599.1, 1477.4, 1448.8, 1316.2, 1226.8, 1108.8, 838.0, 745.2, 720.0, 632.7, 611.9. **HRMS** (DART) m/z: $[C_{34}H_{22}N]^+$, calculated 444.17522, found 444.17347, difference (ppm): -3.95.

9-(4-(pyren-1-yl)phenyl)-9*H*-carbazole-*d₄* (CPP*d₄*)

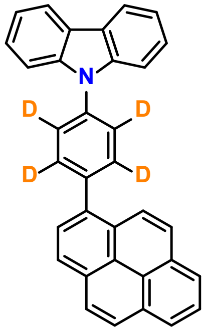

A similar procedure as employed for the synthesis of **CPP** was used with the following quantities of reagents: 500 mg (1.34 mmol, 1 eq.) of 9-(4-iodophenyl)carbazole-d_4 (**1-*d₄***), 395 mg (1.61 mmol, 1.2 eq.) of pyrene-1-boronic acid, 77 mg (0.07 mmol, 0.05 eq.) of tetrakis(triphenylphosphine)palladium(0), and 872 mg (2.68 mmol, 2.0 eq.) of cesium carbonate along with 8 mL of dioxane and 1 mL of deionized H_2O. Purification of the product was carried out similarly as employed for **CPP** (473 mg, **yield 79%**, **m.p.** 225 °C). **^{1}H NMR** (400 MHz, $CDCl_3$) δ: 8.35-8.29 (m, 2H), 8.25-8.20 (m, 4H), 8.15-8.04 (m, 5H), 7.63 (d, *J* = 8.0 Hz, 2H), 7.50 (t, *J* = 7.6 Hz, 2H), 7.35 (t, *J* = 7.6 Hz, 2H). **^{13}C NMR** (100 MHz, $CDCl_3$) δ: 141.0, 140.2, 136.9, 136.8, 131.6, 131.1, 131.0, 128.7, 127.9, 127.8, 127.7, 127.5, 126.2, 126.1, 125.4, 125.2, 125.1, 125.1, 125.0, 124.9, 123.6, 120.5, 120.2, 110.0. **FTIR** (ATR, cm^{-1}) ν: 3045.0, 1922.4, 1895.4, 1597.0, 1488.4, 1449.9, 1307.3, 1226.5, 1108.8, 849.3, 830.0, 745.5, 722.0, 681.1. **HRMS** (DART) m/z: $[C_{34}H_{18}D_4N]^+$, calculated 448.20033, found 448.19908, difference (ppm): -2.78.

Solution ^{1}H and ^{13}C NMR characterization of compounds

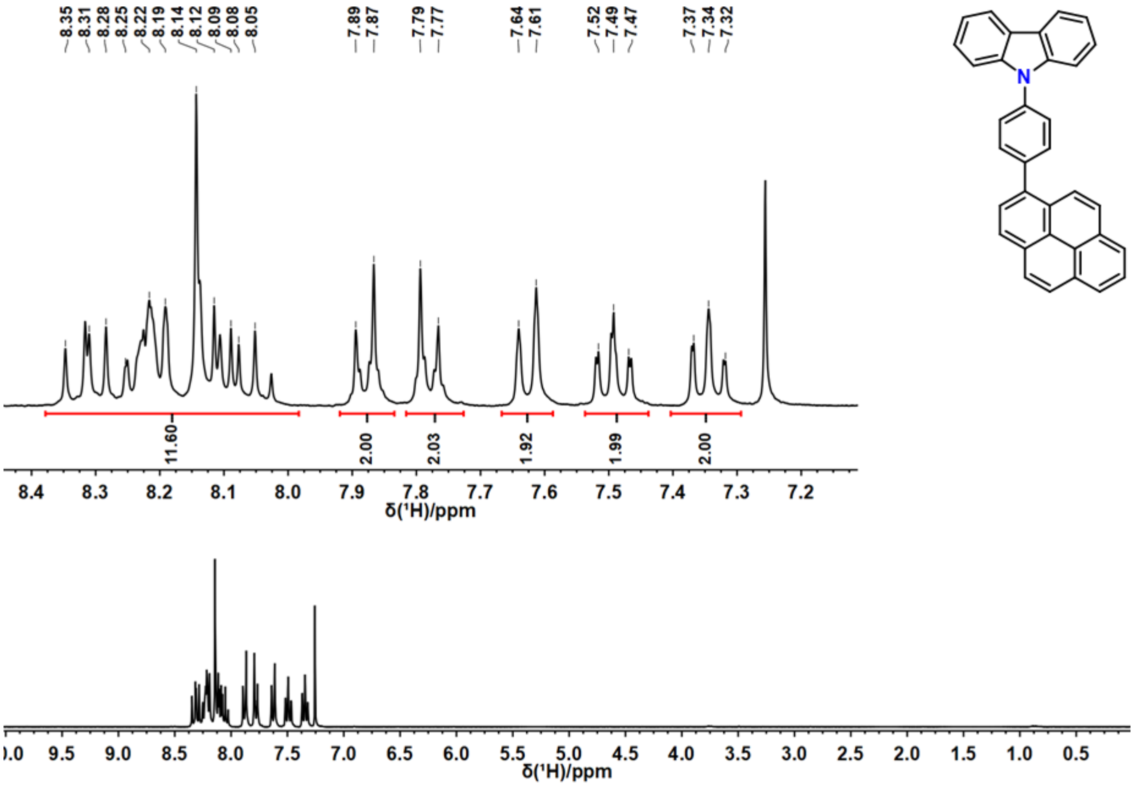

Figure S1. ^{1}H NMR spectra of compound **CPP** ($CDCl_3$, 300 MHz, Room Temperature).

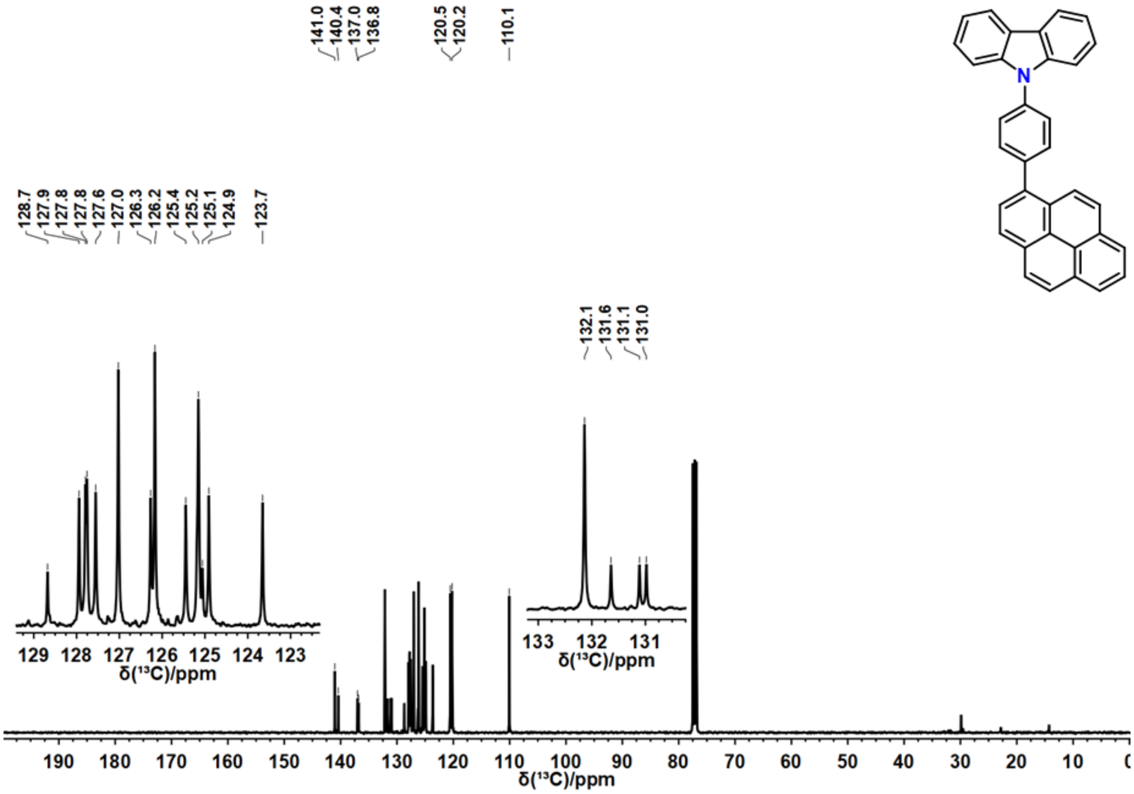

Figure S2. ^{13}C NMR spectra of compound **CPP** ($CDCl_3$, 100 MHz, Room Temperature).

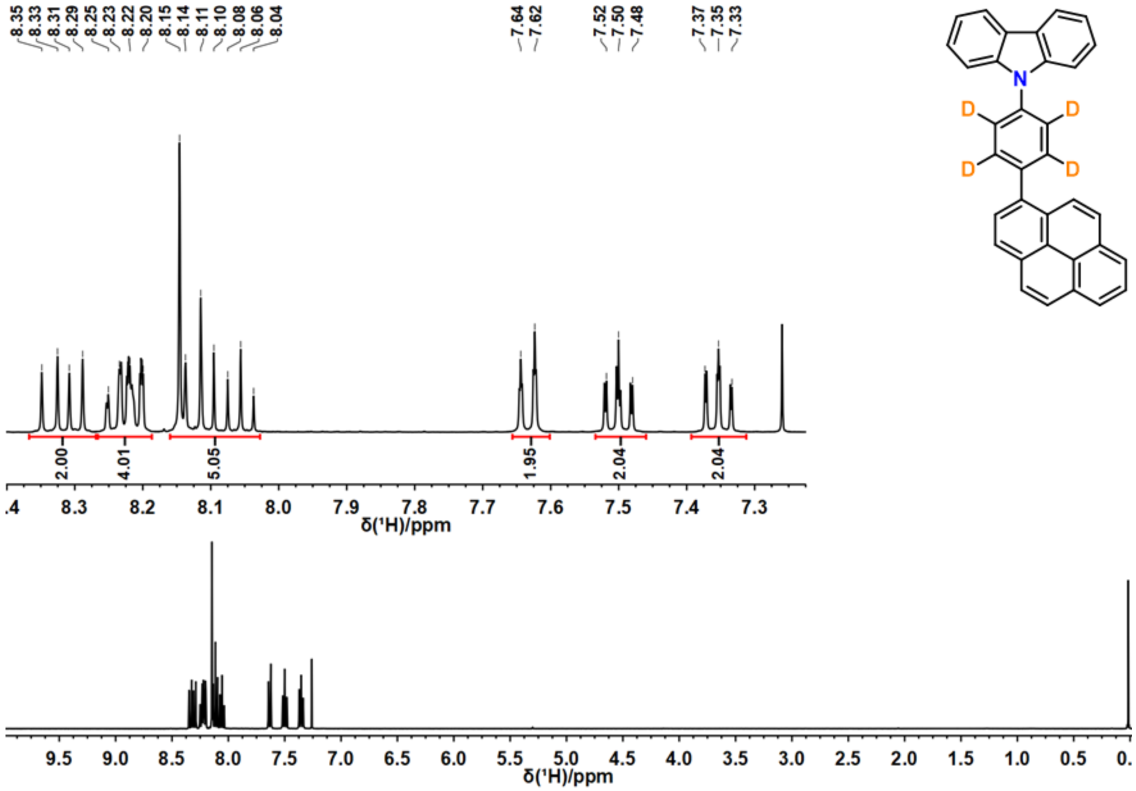

Figure S3. ^{1}H NMR spectra of compound **CPP*d4*** ($CDCl_3$, 400 MHz, Room Temperature)

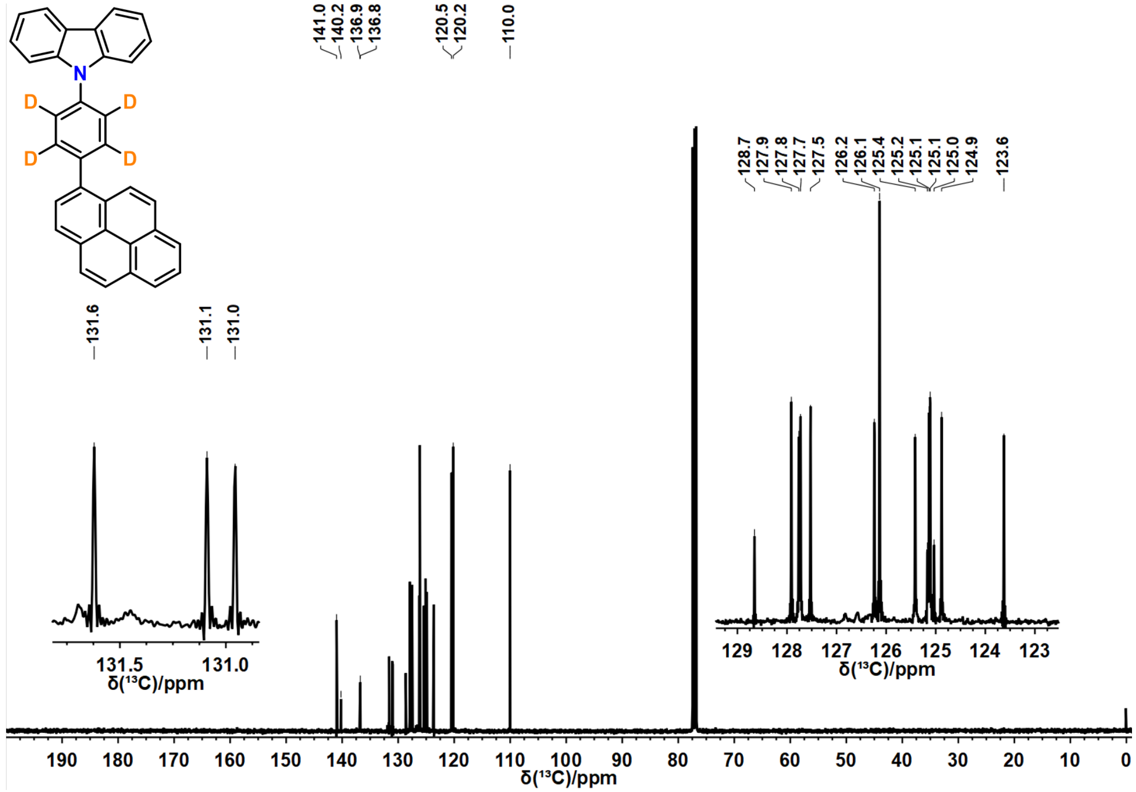

Figure S4. ^{13}C NMR spectra of compound **CPP*d4*** ($CDCl_3$, 100 MHz, Room Temperature)

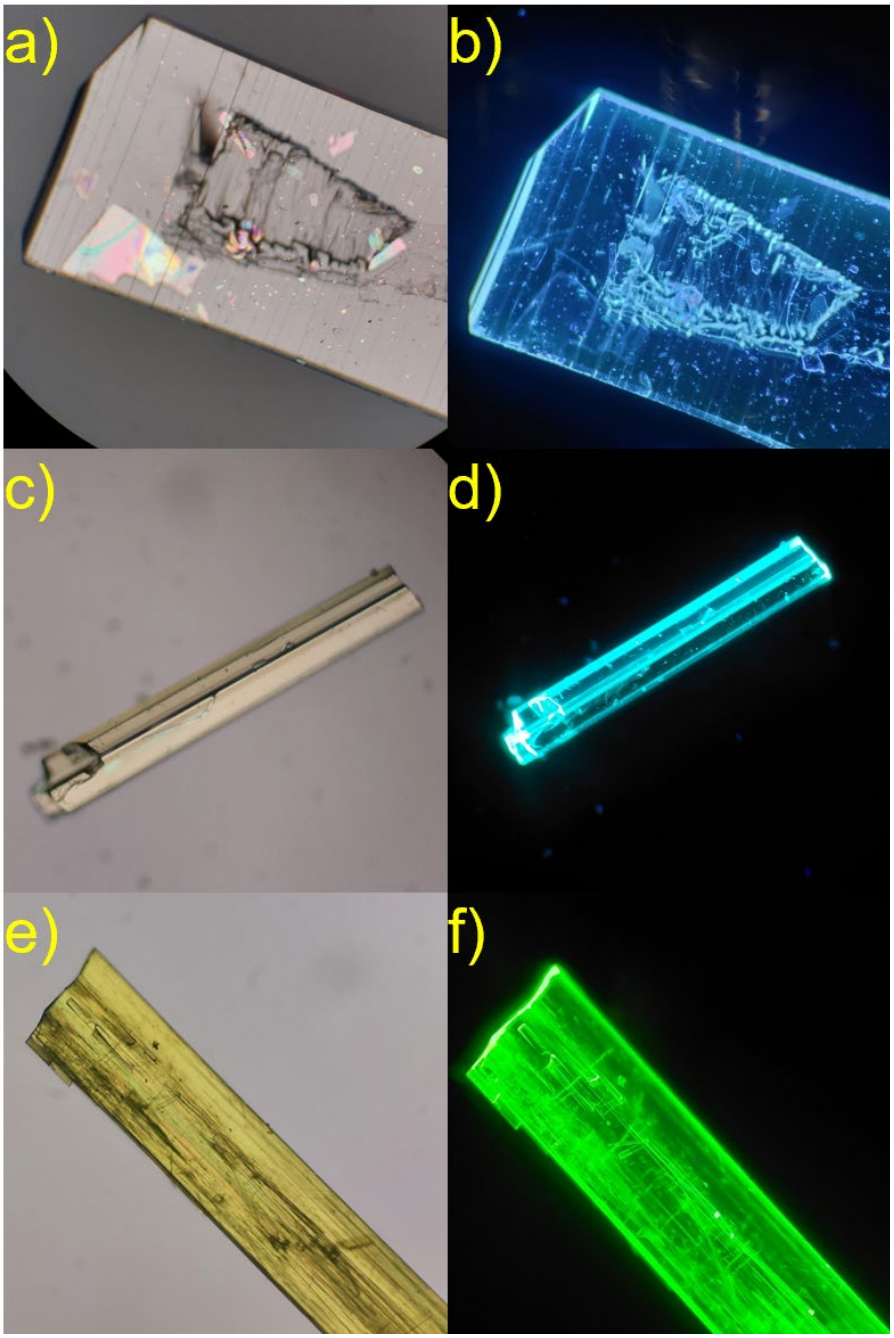

Figure S5. Microscope photographs of the single crystals of starting material CPP (a,b), **1** (c,d), and **2** (e,f) under natural and UV light.

Table S1. Final refinement parameters for **1** at 100, 200 and 300 K.

Identification code	**CPP-TFPN_100K**		**CPP-TFPN_200K**		**CPP-TFPN_300K**	
Empirical formula	$C_{42}H_{21}F_4N_3$		$C_{42}H_{21}F_4N_3$		$C_{42}H_{21}F_4N_3$	
Formula weight	643.62		643.62		643.62	
Temperature [K]	100(2)		200(2)		300(2)	
Wavelength [Å]	0.7288		0.7288		0.7288	
Crystal system	Triclinic		Triclinic		Triclinic	
Space group	P-1		P-1		P-1	
Unit cell dimensions	a = 6.8884(6) Å	α= 88.200(4)°	a = 6.9451(7) Å	α= 88.111(4)°	a = 7.0068(9) Å	α= 87.984(5)°
	b = 9.3805(9) Å	β= 86.613(4)°	b = 9.4266(10) Å	β= 86.132(4)°	b = 9.4964(12) Å	β= 85.422(5)°
	c = 24.113(2) Å	γ = 72.855(3)°	c = 24.197(3) Å	γ = 72.801(4)°	c = 24.289(3) Å	γ = 72.798(5)°
Volume [$Å^3$]	1486.1(2)		1509.7(3)		1538.8(3)	
Z	2		2		2	
Density (calculated) [g cm^{-3}]	1.438		1.416		1.389	
Absorption coefficient [mm^{-1}]	0.107		0.105		0.103	
F(000)	660		660		660	
Crystal size [mm]	0.330 x 0.010 x 0.010		0.330 x 0.010 x 0.010		0.330 x 0.010 x 0.010	
Theta range for data collection [°]	1.735 to 31.434		1.730 to 31.472°		1.725 to 27.195	
Index ranges	$-9 \le h \le 9$<br>$-13 \le k \le 13$<br>$-34 \le l \le 34$		$-9 \le h \le 9$<br>$-13 \le k \le 13$<br>$-34 \le l \le 34$		$-8 \le h \le 8$<br>$-11 \le k \le 11$<br>$-30 \le l \le 30$	
Reflections collected	54172		55223		44571	
Independent reflections	9101 [R(int) = 0.0419]		9187 [R(int) = 0.0433]		6250 [R(int) = 0.0423]	
Completeness to theta = 25.242°	99.8%		98.8%		98.7%	
Absorption correction	Semi-empirical from equivalents		Semi-empirical from equivalents		Semi-empirical from equivalents	
Max. and min. transmission	0.999 and 0.909		0.999 and 0.917		0.999 and 0.926	
Refinement method	Full-matrix least-squares on F^2		Full-matrix least-squares on F^2		Full-matrix least-squares on F^2	
Data / restraints / parameters	9101 / 0 / 442		9187 / 0 / 442		6250 / 0 / 442	
Goodness-of-fit on F^2	1.044		1.066		1.064	
Final R indices [I>2sigma(I)]	R1 = 0.0421<br>wR2 = 0.1154		R1 = 0.0458<br>wR2 = 0.1247		R1 = 0.0474<br>wR2 = 0.1294	
R indices (all data)	R1 = 0.0501<br>wR2 = 0.1212		R1 = 0.0593<br>wR2 = 0.1342		R1 = 0.0625<br>wR2 = 0.1394	
Extinction coefficient	n/a		n/a		n/a	
Largest diff. peak and hole [e$Å^{-3}$]	0.433 and -0.276		0.381 and -0.283		0.298 and -0.235	
CCDC Deposition code	2353024		2353025		2353026	

Table S2. Final refinement parameters for **2** at 100, 200 and 300 K.

Identification code	**CPP-TFTN_100K**		**CPP-TFTN_200K**		**CPP-TFTN_300K**	
Empirical formula	$C_{42}H_{21}F_4N_3$		$C_{42}H_{21}F_4N_3$		$C_{42}H_{21}F_4N_3$	
Formula weight	643.62		643.62		643.62	
Temperature [K]	100(2)		200(2)		298(2)	
Wavelength [Å]	0.71073		0.71073		0.71073	
Crystal system	Triclinic		Triclinic		Triclinic	
Space group	P-1		P-1		P-1	
Unit cell dimensions	a = 6.737(3) Å	α= 85.09(2)°	a = 6.8036(16) Å	α= 85.162(6)°	a = 6.8609(11) Å	α= 85.232(5)°
	b = 9.620(6) Å	β= 84.503(15)°	b = 9.691(2) Å	β= 84.308(6)°	b = 9.7578(17) Å	β= 84.365(4)°
	c = 23.686(12) Å	γ = 75.010(17)°	c = 23.713(6) Å	γ = 74.912(5)°	c = 23.740(4) Å	γ = 74.703(4)°
Volume [Å^3]	1473.0(13)		1499.3(6)		1522.9(4)	
Z	2		2		2	
Density (calculated) [g cm^{-3}]	1.451		1.426		1.404	
Absorption coefficient [mm^{-1}]	0.103		0.102		0.100	
F(000)	660		660		660	
Crystal size [mm]	0.280 x 0.170 x 0.040		0.280 x 0.170 x 0.040		0.280 x 0.170 x 0.040	
Theta range for data collection [°]	2.309 to 26.118		2.296 to 26.730°		2.168 to 26.730	
Index ranges	$-8 \leq h \leq 8$<br>$-11 \leq k \leq 11$<br>$-0 \leq l \leq 29$		$-8 \leq h \leq 8$<br>$-12 \leq k \leq 12$<br>$-0 \leq l \leq 30$		$-8 \leq h \leq 8$<br>$-12 \leq k \leq 12$<br>$-0 \leq l \leq 30$	
Reflections collected	5817		6357		6454	
Independent reflections	5817 [R(int) = 0.2225]		6357 [R(int) = 0.5651]		6454 [R(int) = 0.4279]	
Completeness to theta = 25.242°	99.6%		99.7%		99.8%	
Absorption correction	Semi-empirical from equivalents		Semi-empirical from equivalents		Semi-empirical from equivalents	
Max. and min. transmission	0.7454 and 0.4949		0.7454 and 0.5919		0.7454 and 0.6298	
Refinement method	Full-matrix least-squares on F^2		Full-matrix least-squares on F^2		Full-matrix least-squares on F^2	
Data / restraints / parameters	5817 / 753 / 444		6357 / 753 / 444		6454 / 753 / 444	
Goodness-of-fit on F^2	1.185		1.123		1.083	
Final R indices [I>2sigma(I)]	R1 = 0.1283<br>wR2 = 0.2945		R1 = 0.1662<br>wR2 = 0.3280		R1 = 0.1332<br>wR2 = 0.2787	
R indices (all data)	R1 = 0.1766<br>wR2 = 0.3148		R1 = 0.3165<br>wR2 = 0.3883		R1 = 0.2899<br>wR2 = 0.3414	
Extinction coefficient	0.037(5)		0.068(8)		0.044(7)	
Largest diff. peak and hole [eÅ^{-3}]	0.675 and -0.603		0.598 and -0.706		0.544 and -0.454	
CCDC Deposition code	2353027		2353028		2353029	

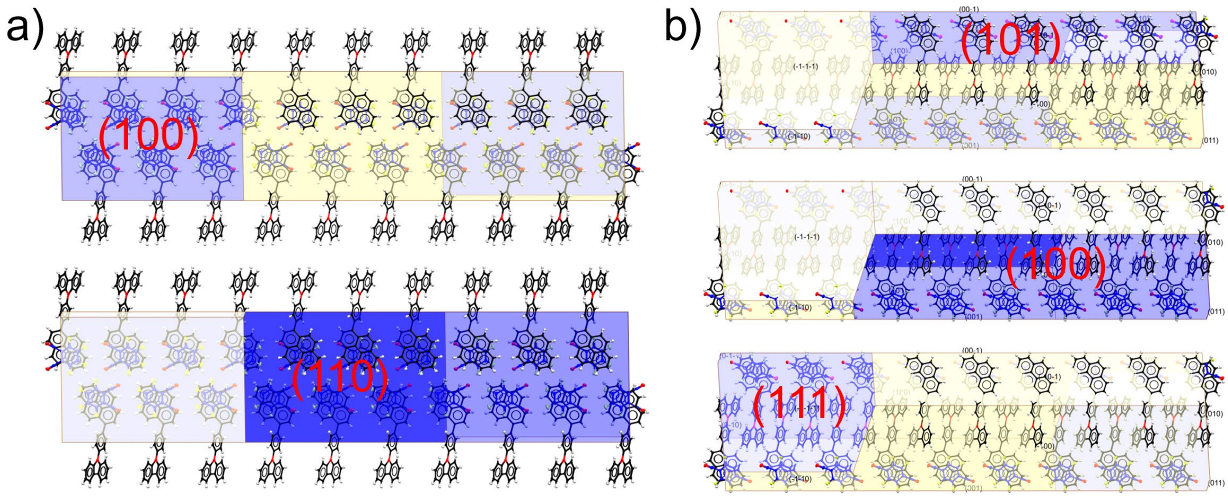

Figure S6. Simulated morphology of cocrystals a) **1** and b) **2**, using the Materials Studio software. The blue-shadowed parts depict the facets with the lowest attachment energies, which correspond to the most probable zones of propagation during the crystal growth. The thermal ellipsoids in the morphologies were represented at 50% level probability.

Table S3. Calculated attachment energies of facets in the crystalline habits of the cocrystals

Cocrystal	Facet	Multiplicity	d_{hkl} (Å)	E_{att} (kcal/mol)	Total facet area (%)
**1**	(100)	2	6.573	-77.279	7.55
	(110)	2	6.247	-85.436	3.62
**2**	(101)	2	6.382	-78.923	3.12
	(100)	2	6.489	-79.412	5.22
	(111)	2	6.062	-85.554	3.32

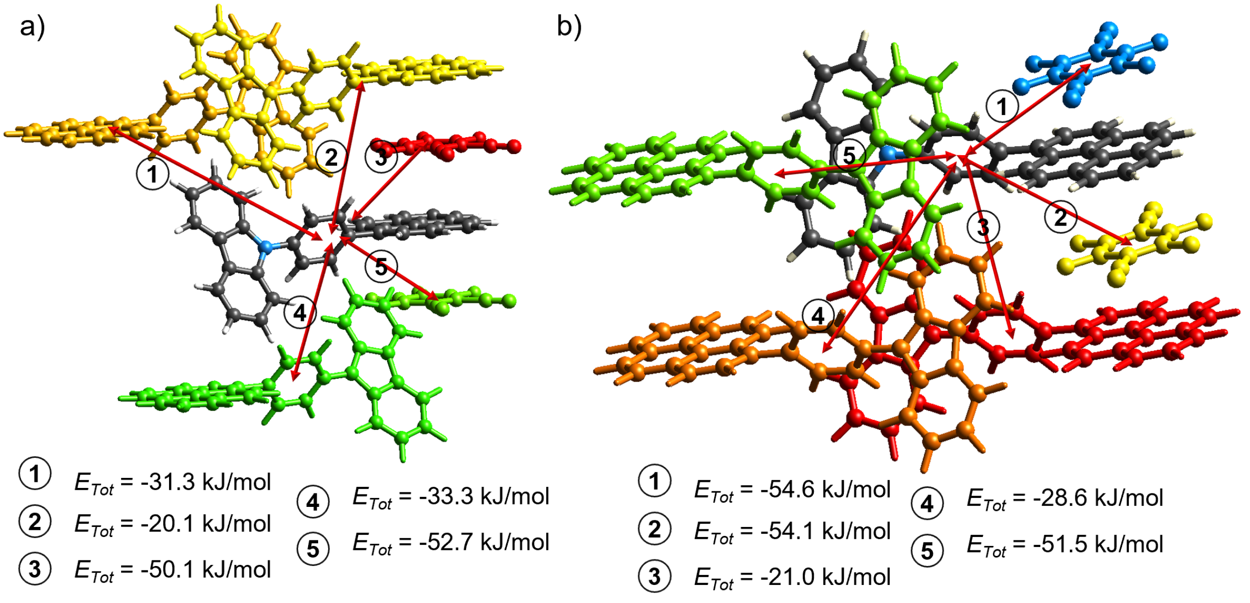

Figure S7. Energy interactions between dyads of molecules inside the crystalline packing of a) **1** and b) **2**.

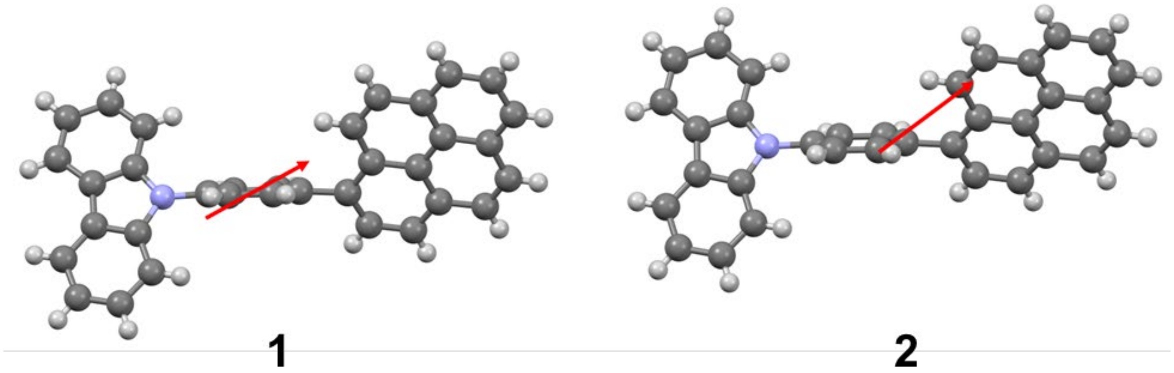

Figure S8. Vectorial orientation of the Transition Dipole Moments calculated for CPP molecules in the cocrystals.

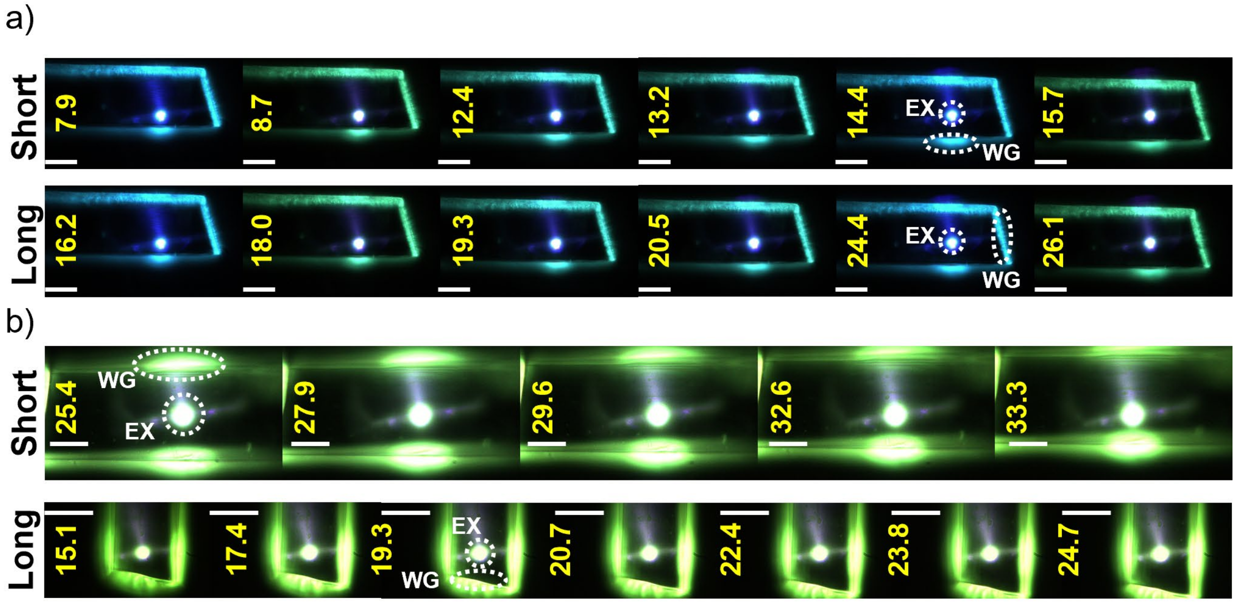

Figure S9. Micro-photoluminescence images of OWG measurements in cocrystals a) **1** and b) **2** with a lase excitation λ = 405 nm, applied at different positions along the short and long axis directions. The numbers inside each picture is the distance (*D*) between the excitation (EX) and detection (WG) positions.

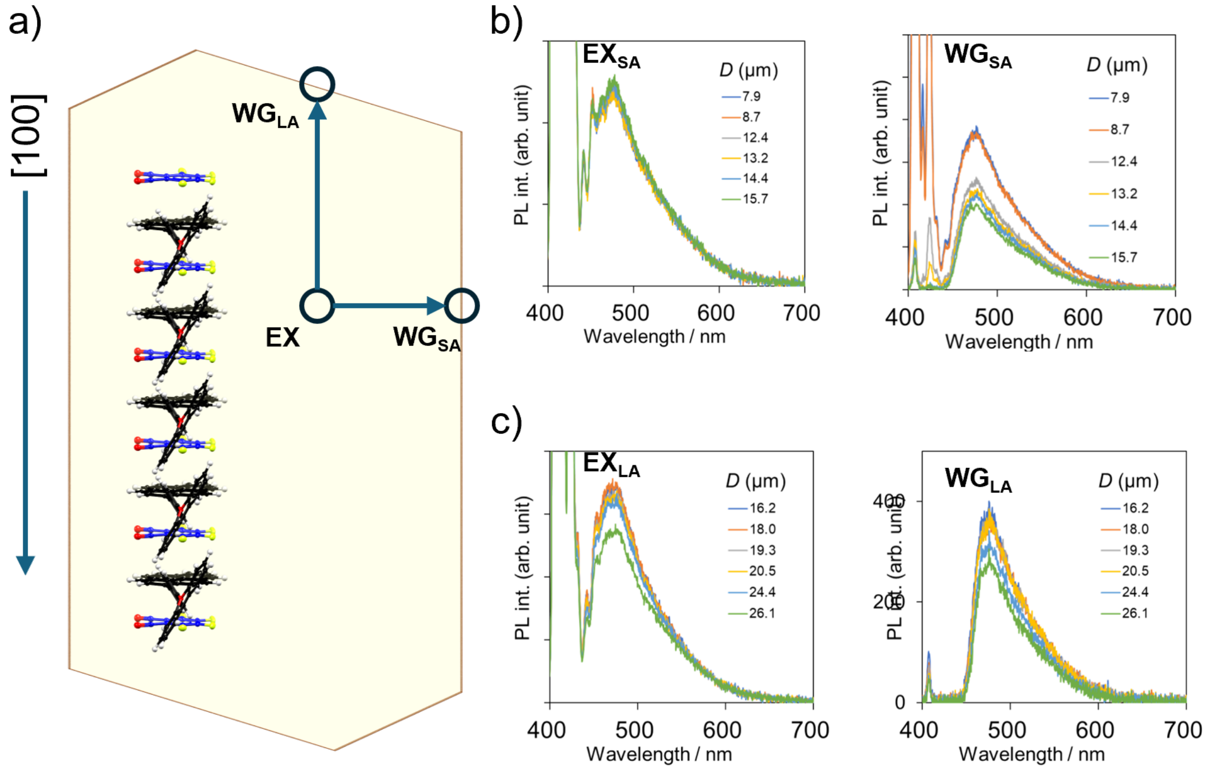

Figure S10. a) Schematics of the optical waveguiding measurements on the single crystal of **1** over the short and long axes; micro-photoluminescence spectra measured at the excitation (EX) and waveguided (WG) positions b) in the short, and c) long-axis.

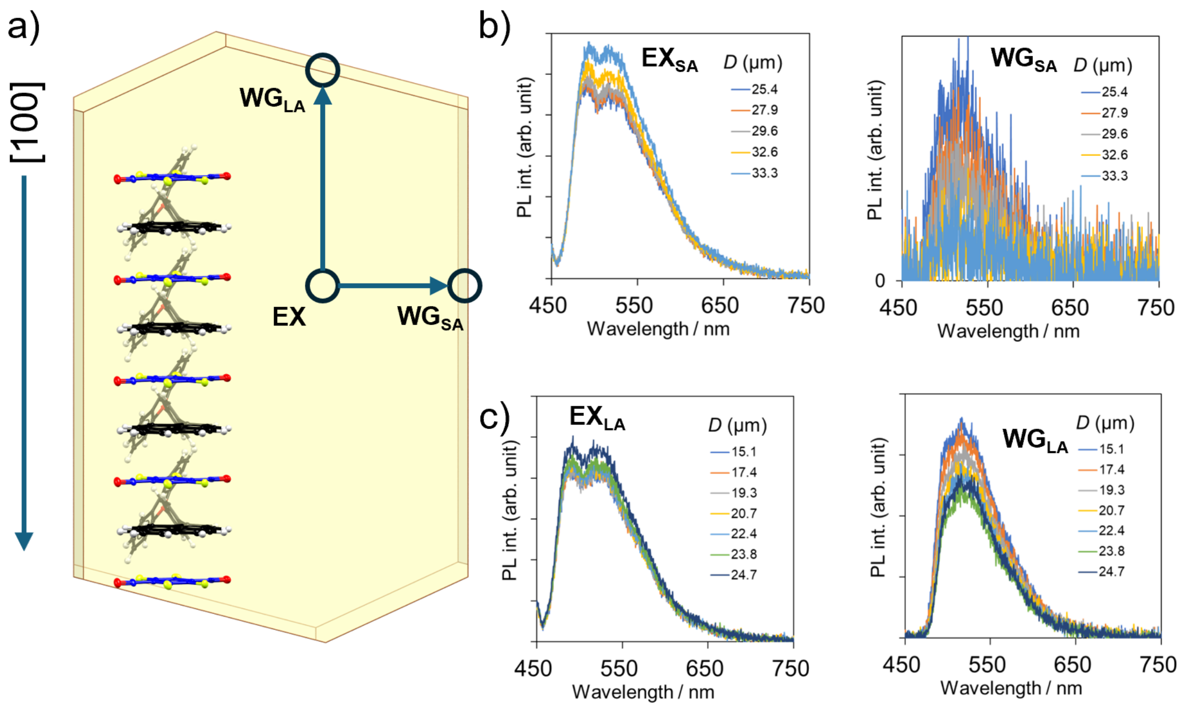

Figure S11. a) Schematics of the optical waveguiding measurements on the single crystal of **2** over the short and long axes; micro-photoluminescence spectra measured at the excitation (EX) and waveguided (WG) positions b) in the short, and c) long-axis.

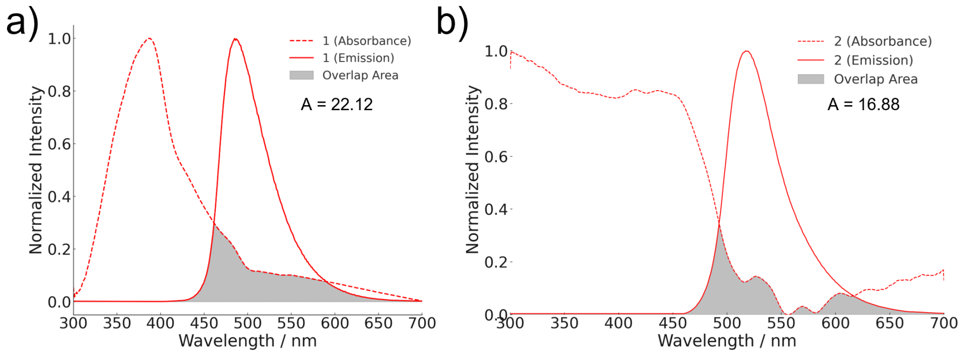

Figure S12. Overlapped absorbance and emission spectra for cocrystals. The resulting areas are highlighted in each panel.

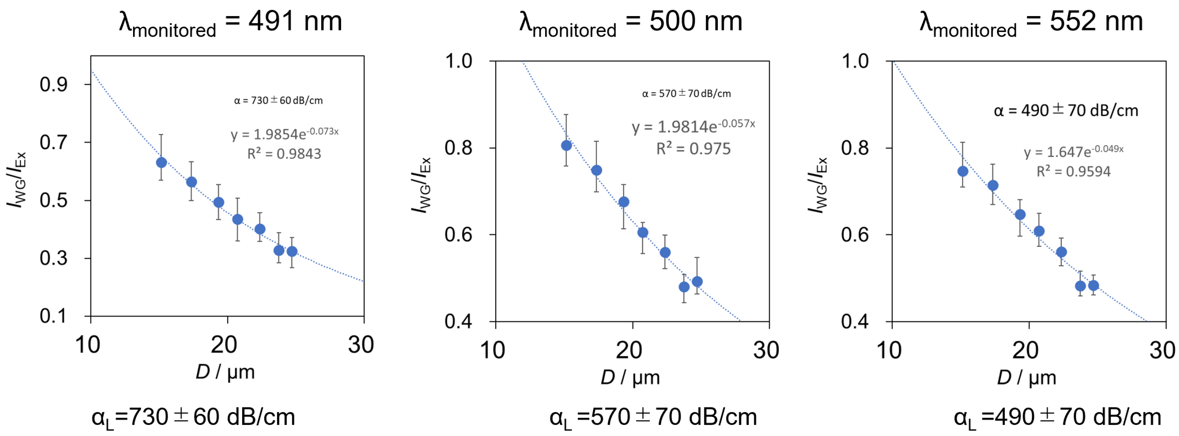

Figure S13. Measurements of optical loss coefficient along the long-axis in cocrystal **2** under different laser wavelengths.

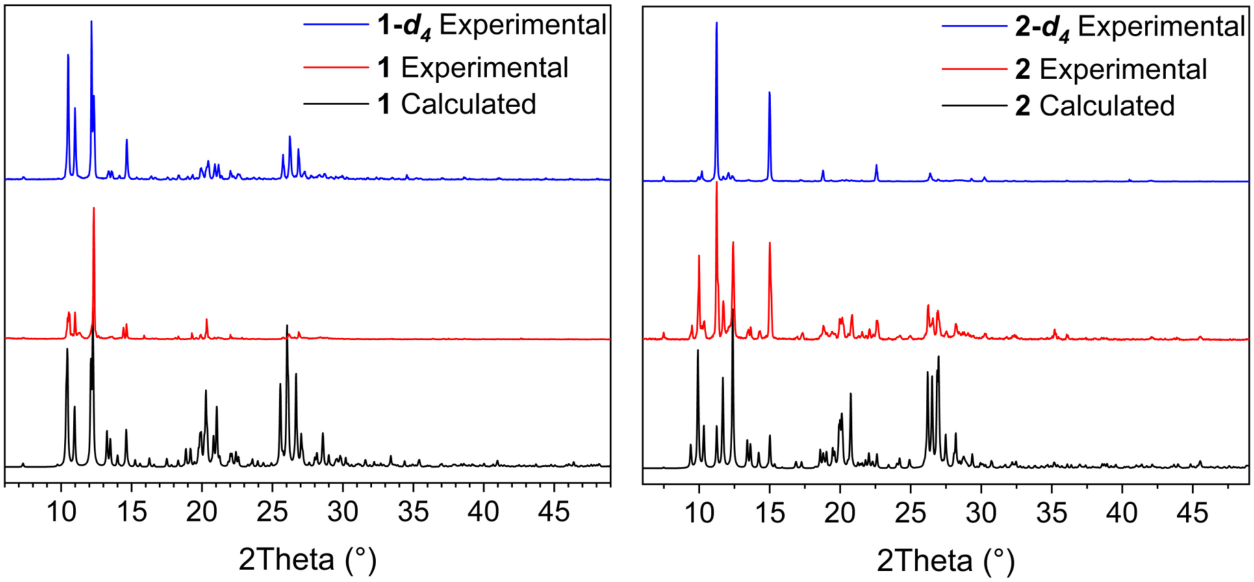

Figure S14. PXRD patterns of the solids herein reported.

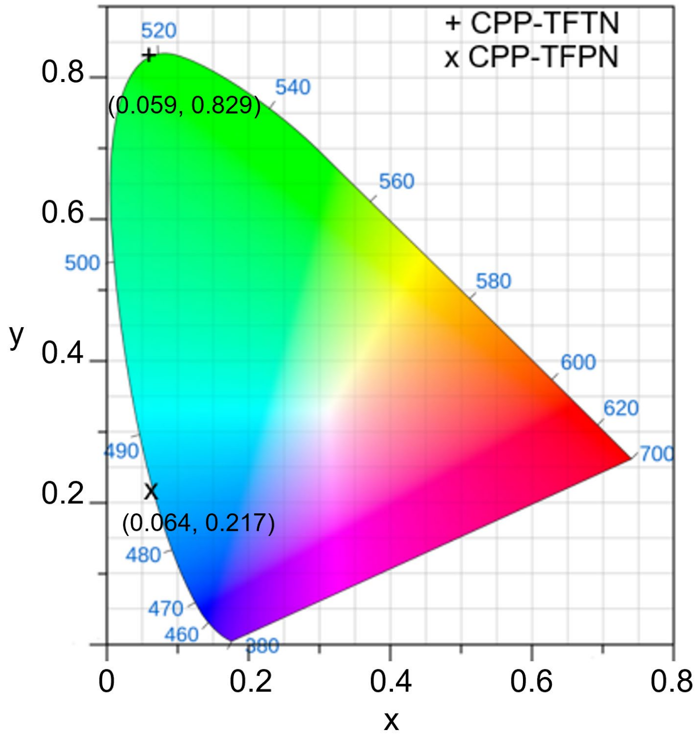

Figure S15. 1931 CIE (x,y) color space chromaticity diagram with the coordinates of the converted fluorescence emission wavelengths of the cocrystals.

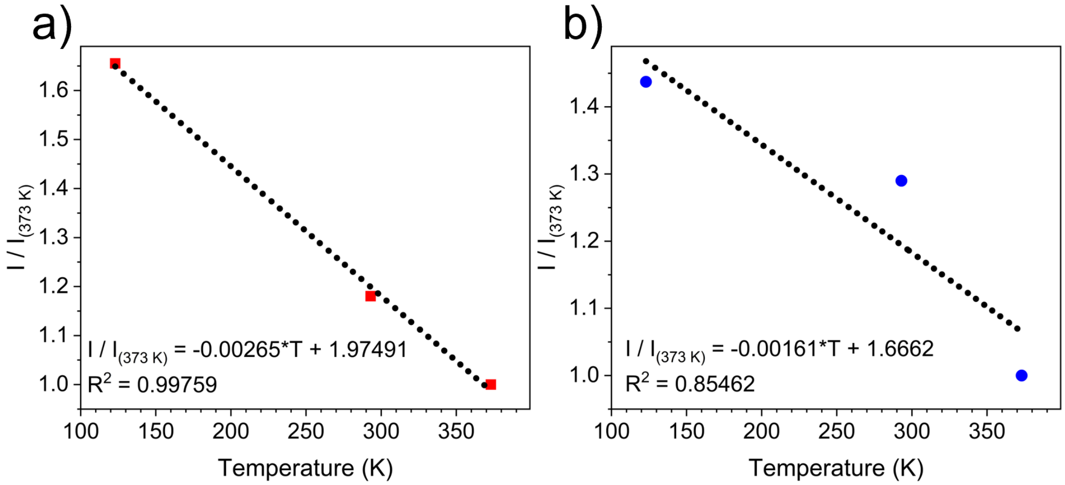

Figure S16. Dependence of the temperature in the photoluminescence intensity respect to the measurement at 373 K for a) cocrystal **1** and b) cocrystal **2**.

Table S4. Summary of representative values for the tail fitting of the time-resolved fluorescence decay curves.

Cocrystal	Temperature (K)	λ_{ex} (nm)	λ_{em} (nm)	τ_1 (ns)	Rel $_1$ (%)	τ_2 (ns)	Rel $_2$ (%)	τ_3 (ns)	Rel $_3$ (%)	τ_{avg} (ns)	$\chi 2$
**1**	173	375	492	23.13	68.82	51.54	31.18			31.99	1.01
	293	375	492	26.28	89.57	51.46	10.43			28.91	1.08
	373	375	492	25.11	93.99	51.46	6.01			26.69	0.98
**2**	173	375	496	24.37	10.09	65.06	89.10	317.28	0.80	62.97	1.00
	173	375	526	35.74	11.28	67.87	87.75	616.72	0.97	69.56	1.00
	293	375	518	28.86	10.27	59.87	88.74	792.94	0.98	63.90	1.04
	373	375	518	24.91	7.42	57.23	91.65	792.00	0.93	61.70	1.08

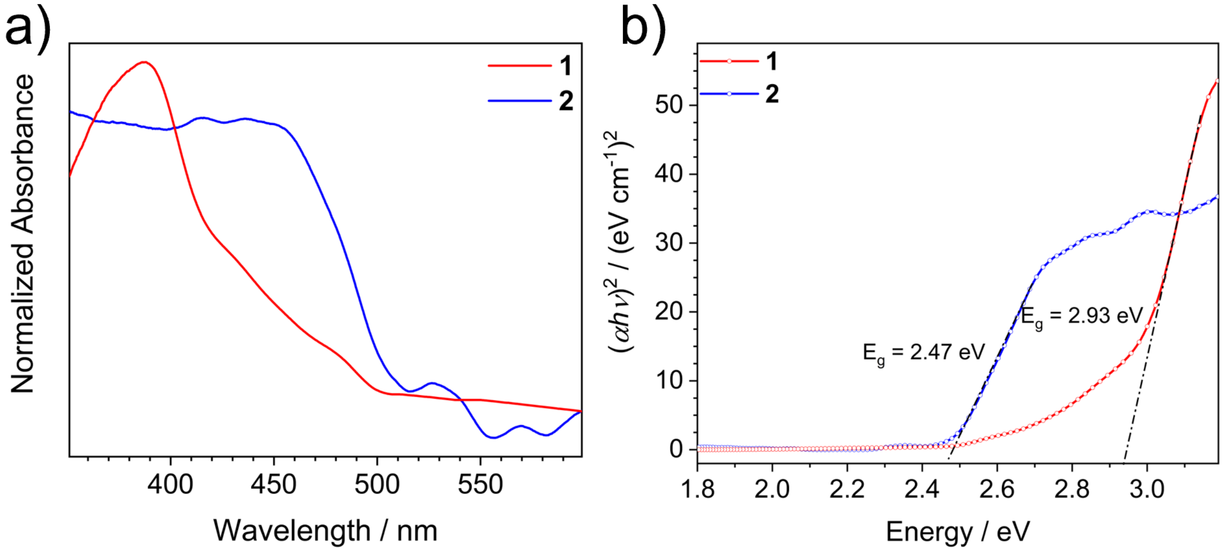

Figure S17. a) Solid-state UV-Vis spectra of the cocrystals using the diffuse reflectance technique; b) Modified Tauc plots obtained from the diffuse reflectance data, displaying the electronic optical gap (E_g) for each cocrystal.

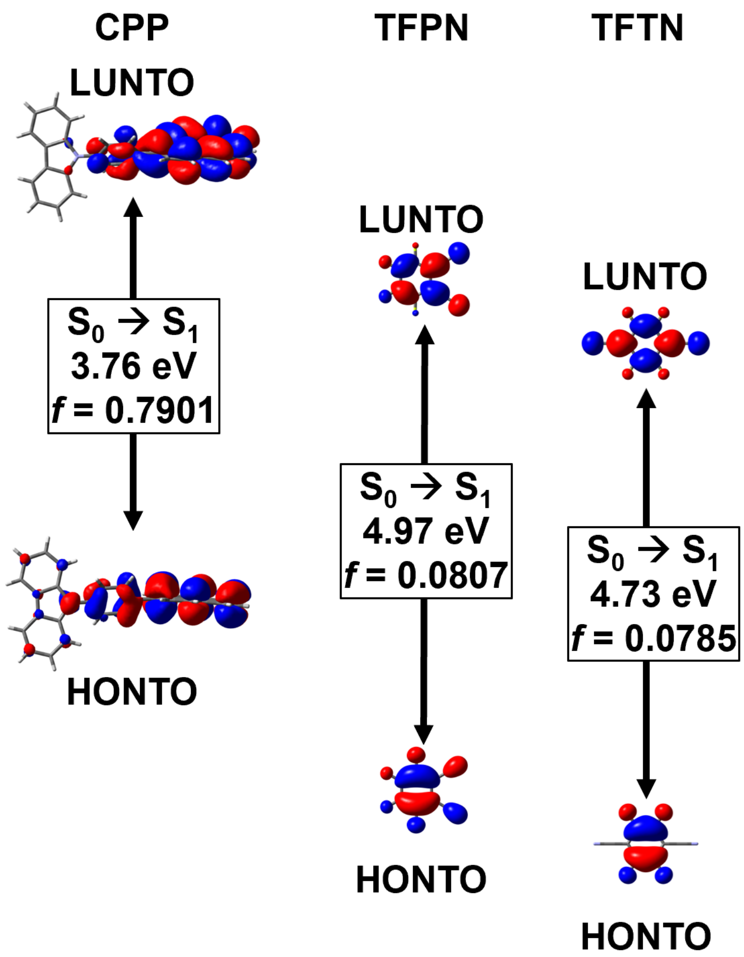

Figure S18. Natural Transition Orbital (NTOs) of starting materials **CPP**, **TFPN** and **TFTN** calculated at the ground state with the M06-2X functional at the 6-311G(d,p) level of theory. The associated energies (in eV) and oscillator strengths (*f*) are included.

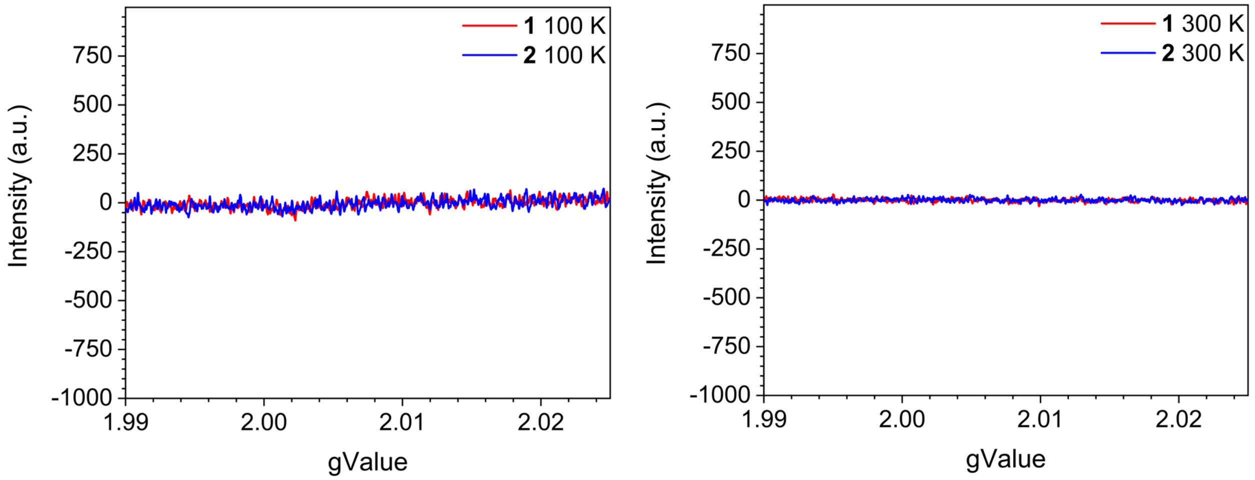

Figure S19. Solid-state Electron-Spin Resonance spectra of cocrystals presented in this work, at 100 and 300 K.

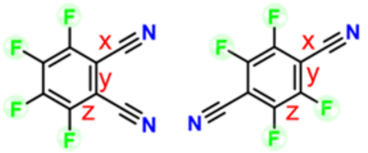

$$\rho = \frac{1}{2}\left[\left(1 - \frac{x_{CT} - y_{CT}}{x_N - y_N}\right) + \left(1 - \frac{z_{CT} - y_{CT}}{z_N - y_N}\right)\right]$$

The degree of charge-transfer, denoted by ρ, is a measurement of the affinity between D:A dyads and provide valuable insights for conductivity and the photophysical behavior of the crystalline solids. A method for the calculation of this value relies on the SC XRD structures of the starting materials (acceptors TFPN and TFTN) and the cocrystals. The important distances, which are prone to change due to the re-distribution of the electron density, are highlighted in red letters in the structures above. Moreover, the labels '*CT*' and '*N*' are the distances in the charge transfer cocrystal and neutral forms, respectively. The value is normalized between $0 \leq \rho \leq 1$, being 0 an inexistent CT phenomenon, whereas 1 is a highly ionic character. The Table S5 contains the complete data set for the distances of both starting materials and cocrystals.

Table S5. Measured relevant bond distances in the SC XRD structures of the starting materials and cocrystals for the determination of the degree of charge transfer.

Distances	TFPN	TFTN
x_{CT} (Å)	1.414	1.418
x_N (Å)	1.428	1.424
y_{CT} (Å)	1.395	1.393
y_N (Å)	1.405	1.408
z_{CT} (Å)	1.363	1.388
z_N (Å)	1.376	1.387
**ρ (e)**	**0.035**	**0.100**

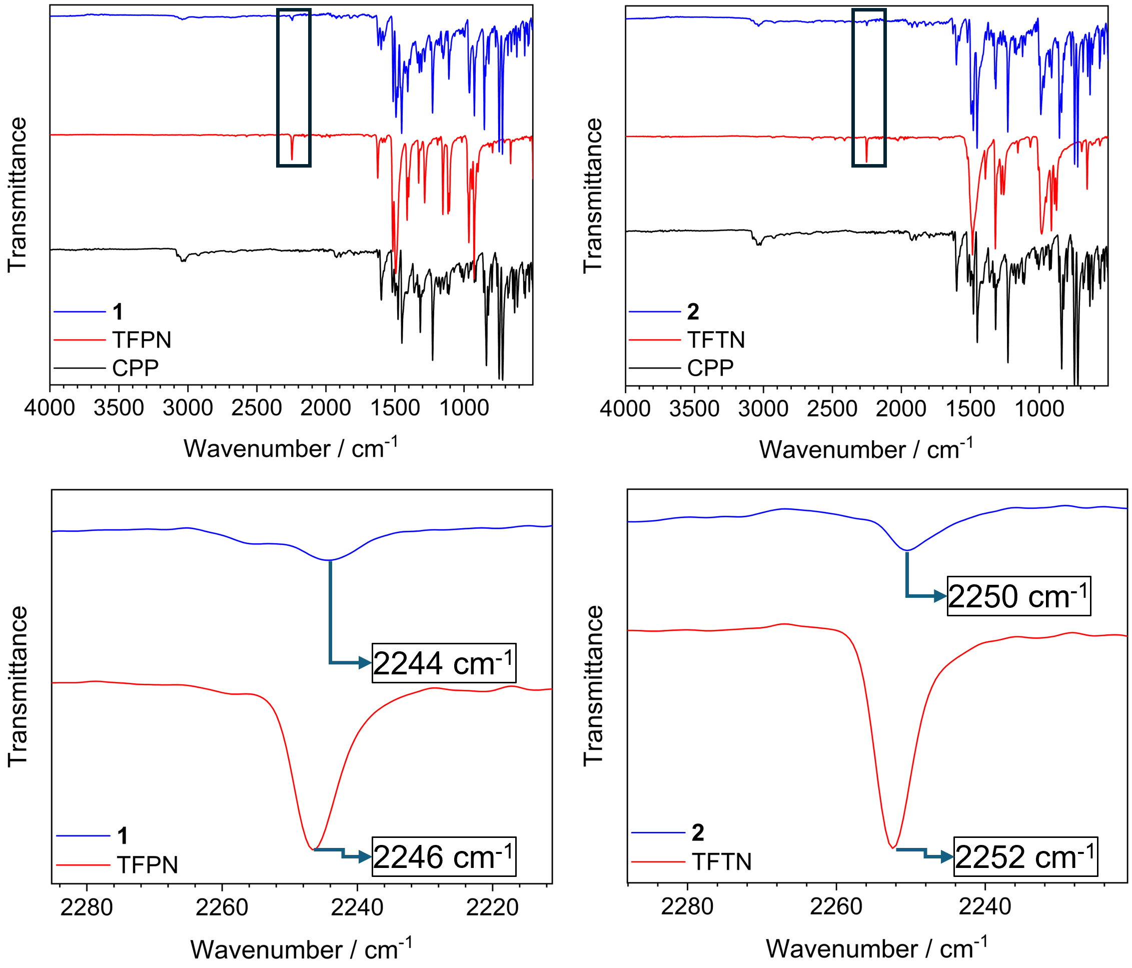

Figure S20. Fourier-Transformed Infrared spectra of the cocrystals. The spectrum at the bottom is a close-up corresponding to the C≡N stretching band of cyano groups in the starting materials and cocrystals.

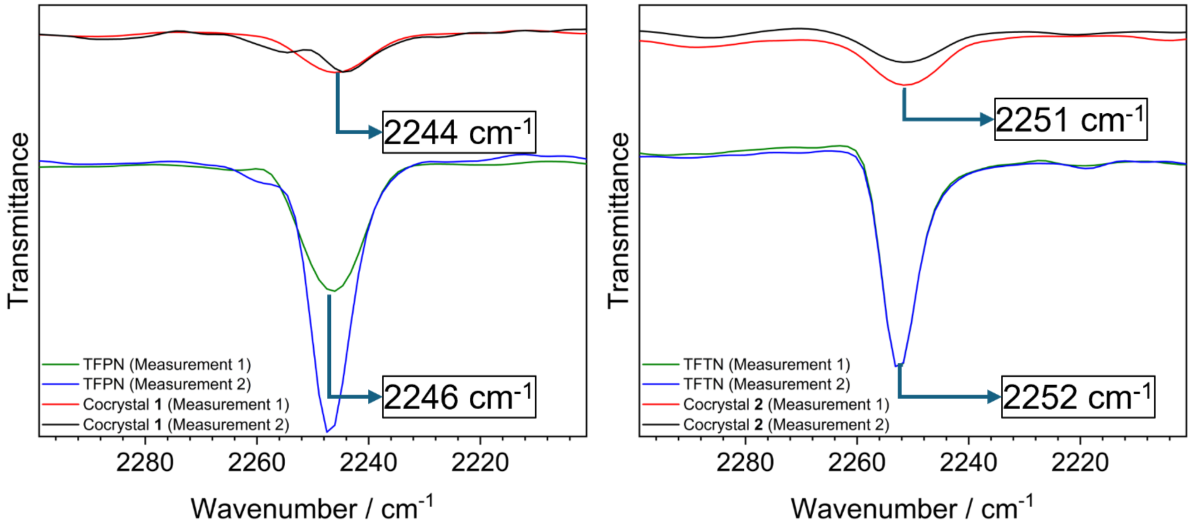

Figure S21. Fourier-Transformed Infrared spectra of the cocrystals to ensure reproducibility of the wavenumber shift due to the low degree of intermolecular charge-transfer. The spectra display a close-up of the C≡N stretching band of cyano groups in the starting materials and cocrystals.

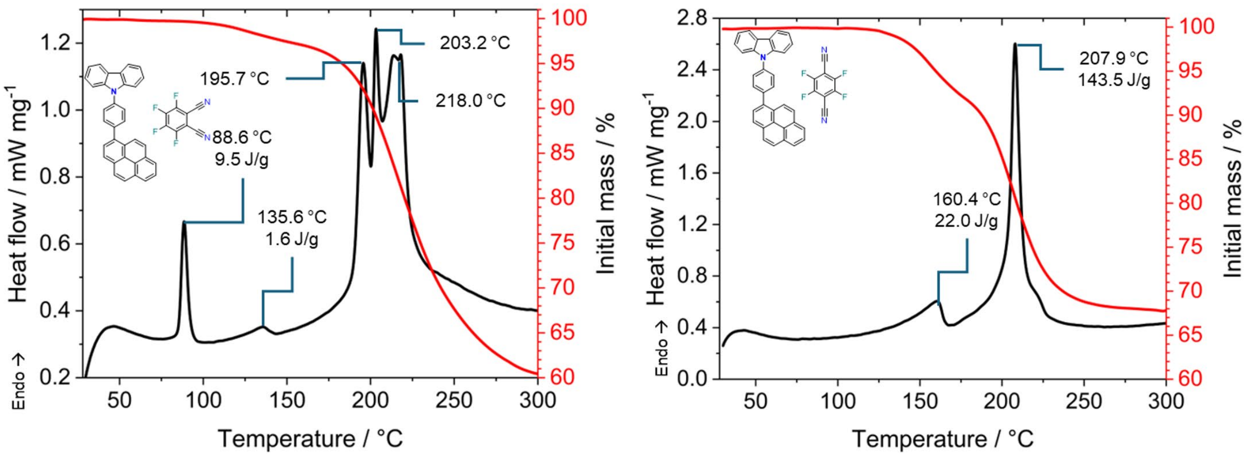

Figure S22. Coupled TGA and DSC analysis of cocrystals.

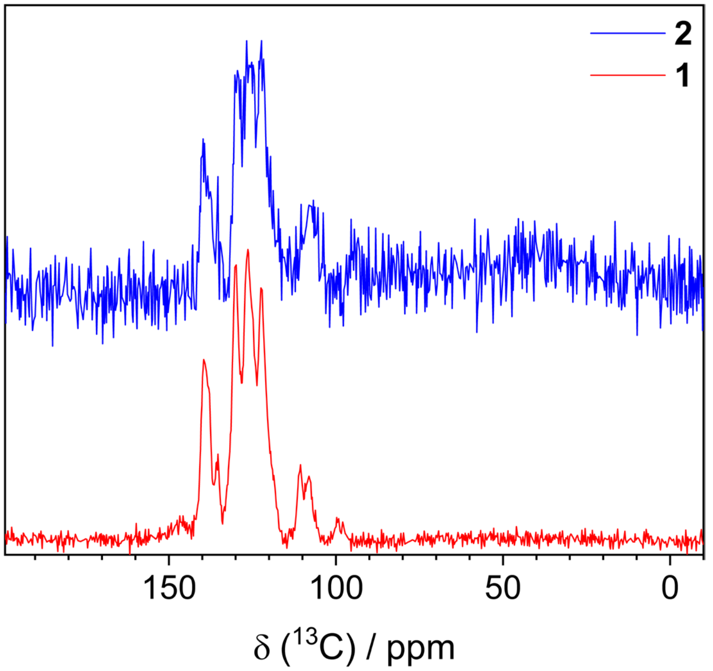

Figure S23. Solid-state Nuclear Magnetic Resonance through the ^{13}C Cross-Polarization Magic-Angle Spinning (CP MAS) spectra at room temperature of the cocrystals reported.

Table S6. Measured T_1 ^{19}F relaxation times through saturation-recovery experiments in powdered samples in ssNMR.

<table>
<tr><th colspan="2">Cocrystal 1</th><th colspan="2">Cocrystal 2</th></tr>
<tr><th>Corrected Temperature (K)</th><th>T₁ (s)</th><th>Corrected Temperature (K)</th><th>T₁ (s)</th></tr>
<tr><td>313</td><td>7.8</td><td rowspan="2">313</td><td rowspan="2">834.8</td></tr>
<tr><td>299</td><td>9.1</td></tr>
<tr><td>284</td><td>10.8</td><td rowspan="2">299</td><td rowspan="2">913.7</td></tr>
<tr><td>265</td><td>13.2</td></tr>
<tr><td>245</td><td>17.8</td><td rowspan="2">284</td><td rowspan="2">1014.8</td></tr>
<tr><td>229</td><td>22.2</td></tr>
</table>

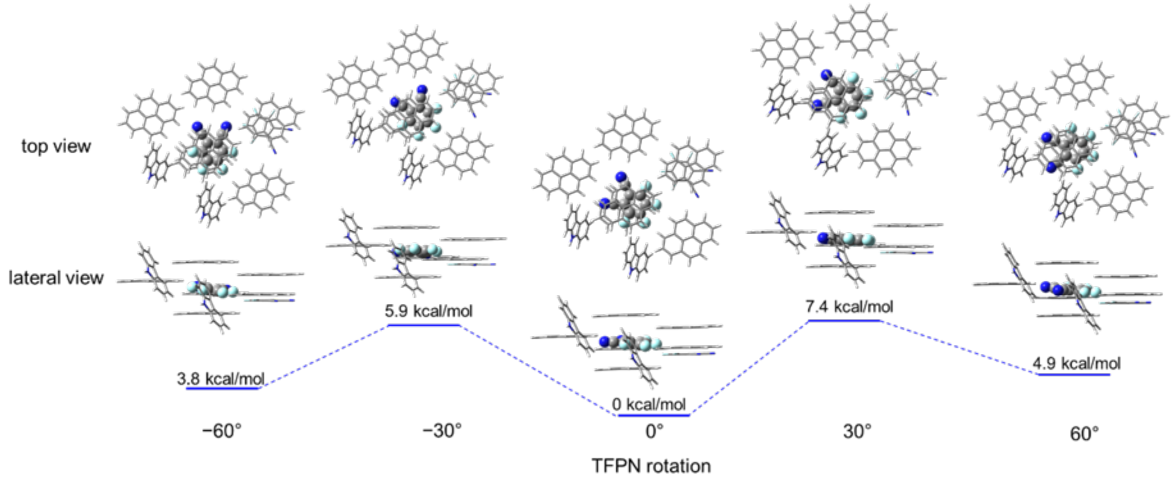

Figure S24. Energetic rotational profile calculated for a fragment of cocrystal **1**.

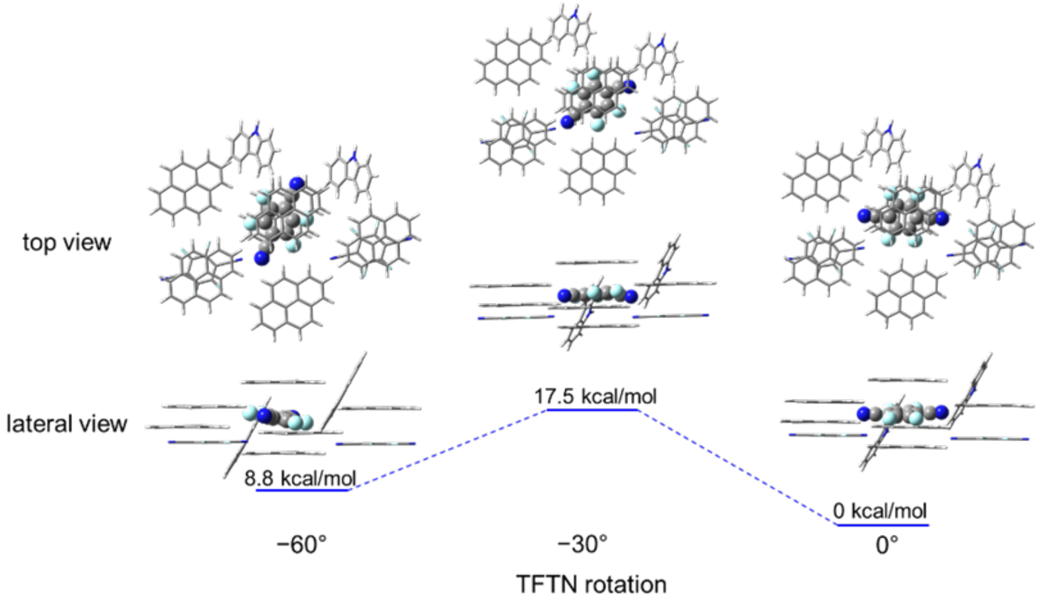

Figure S25. Energetic rotational profile calculated for a fragment of cocrystal **2**.

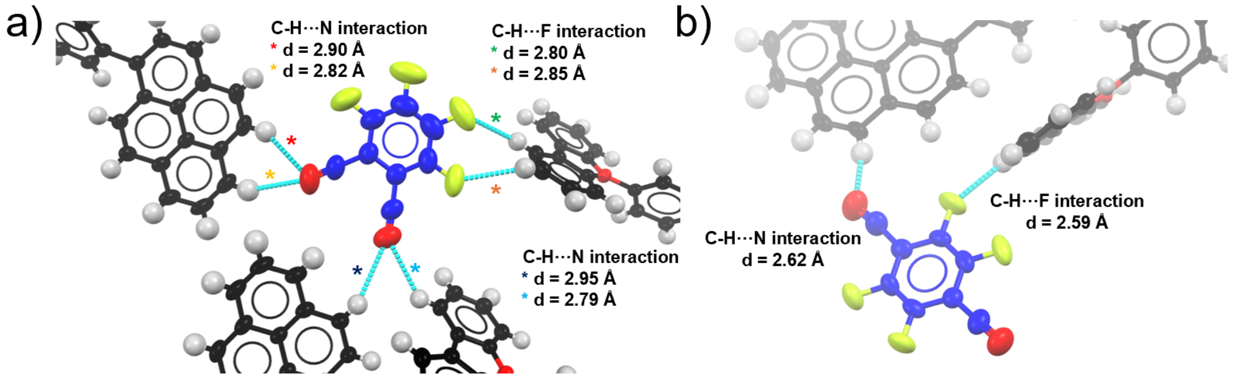

Figure S26. Analysis and schemes of the non-covalent interactions around the fluorinated fragments of the CPP cocrystals herein described.

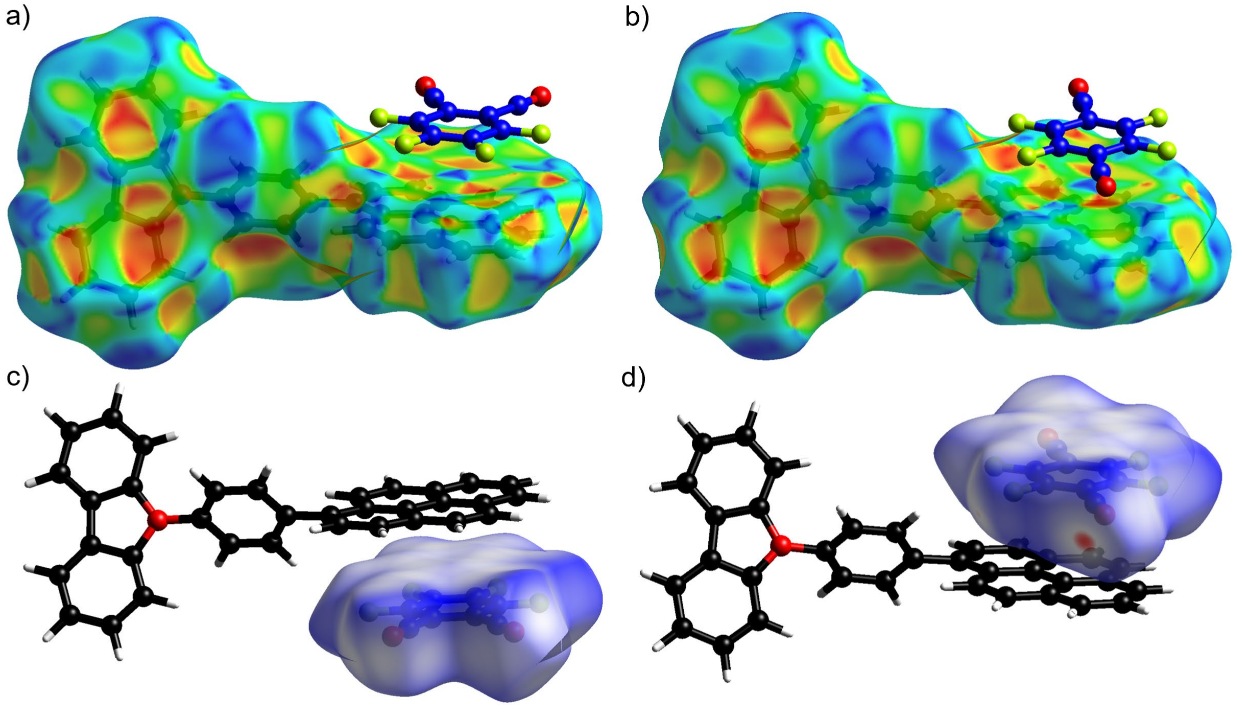

Figure S27. a,b) Hirshfeld surfaces centered on **CPP** for both cocrystals plotted with shape index, illustrating the deformation of the surface over the pyrene fragment due to the π-stacking with acceptors; c,d) Hirsfeld surfaces centered on acceptors TFPN and TFTN, respectively, depicting the influence of non-covalent interactions around these fragments as hotspots (red).

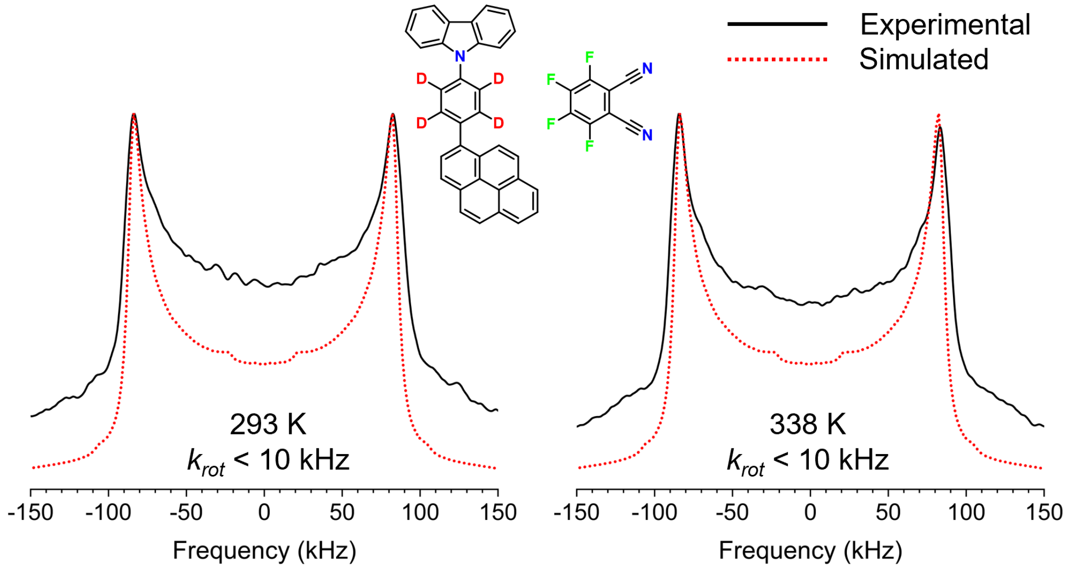

Figure S28. ssNMR Variable Temperature ^{2}H quadrupolar spin-echo spectra of microcrystalline samples of **1-*d*$_4$**. The black and red lines highlight the experimental and simulated spectra, respectively.

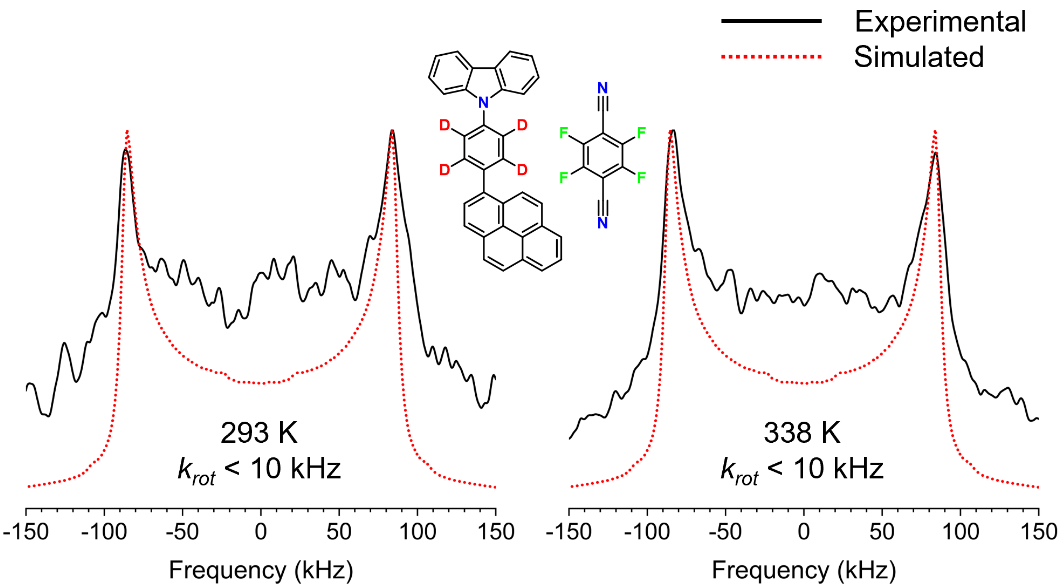

Figure S29. ssNMR Variable Temperature ^{2}H quadrupolar spin-echo spectra of microcrystalline samples of **2-*d*$_4$**. The black and red lines highlight the experimental and simulated spectra, respectively.

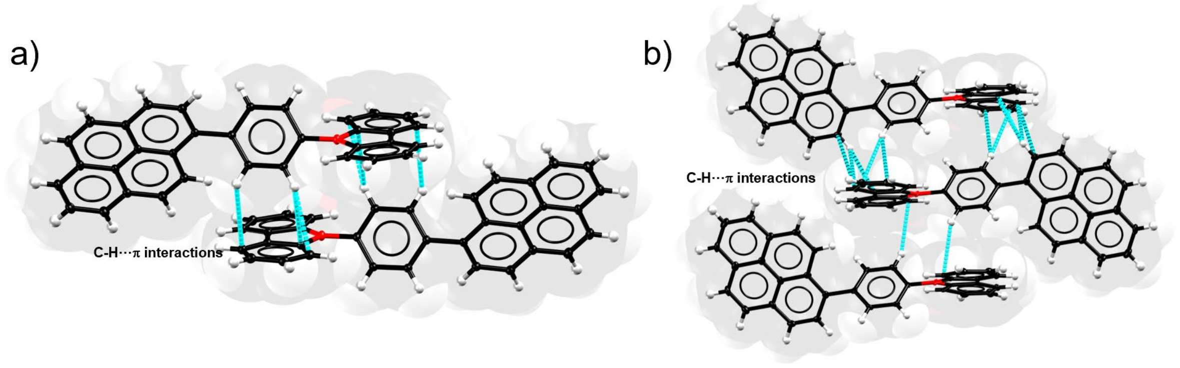

Figure S30. Non-Covalent Interactions (NCI) surrounding the phenylene fragment in CPP molecules of a) cocrystal **1** and b) cocrystal **2**. Thermal ellipsoids were drawn at 50% level of probability.

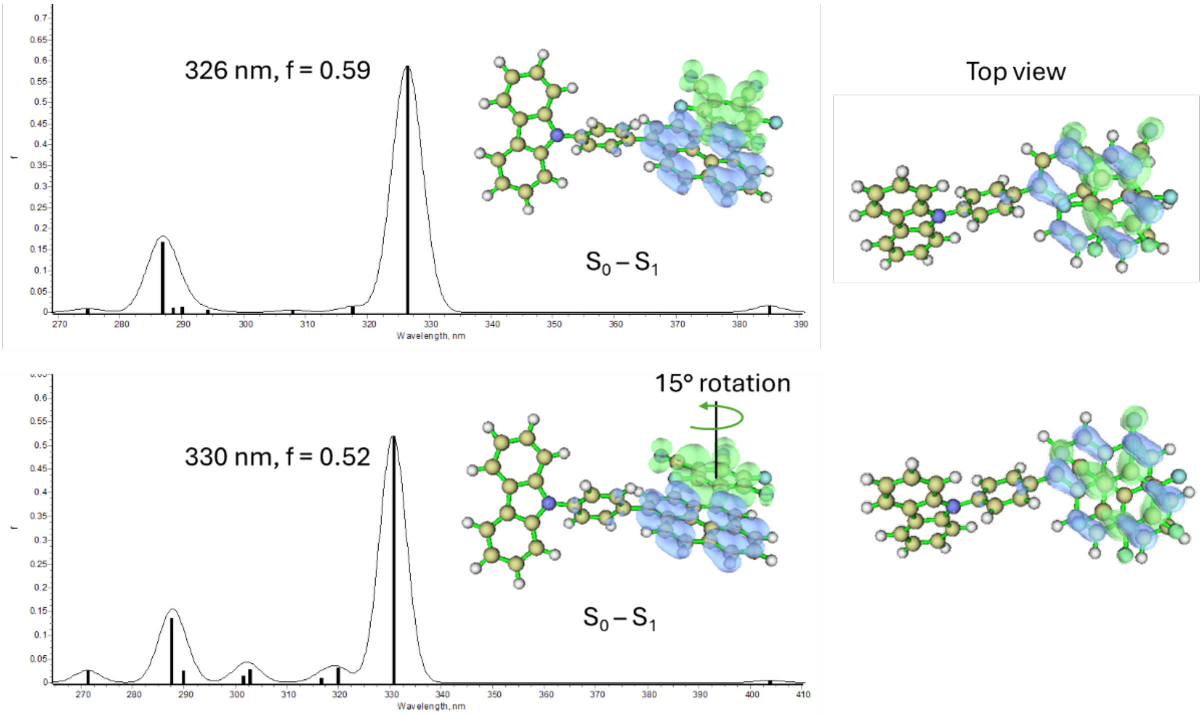

Figure S31. Simulated UV/vis absorption spectra (black line) with oscillator strengths (black sticks) of the donor-acceptor geometry taken from the cocrystal **1** (top) and a modeled structure (bottom) with the molecule of TFPN rotated by 15º from the initial geometry, based on the singlet state TD-DFT calculations [M06-2X/6-311G(d,p)]. Illustration of the contributed hole (green) and electron donor (blue) orbitals of the donor-acceptor structures.

References

[S1] Aguilar-Granda, A., García-González, M. C., Pérez-Estrada, S., Kozina, A., Rodríguez-Molina, B., *J. Phys. Chem. C,* **2018**, *122*, 27093-27099.

[S2] Bruker. (2006b). SAINT, Version 8.38 (Bruker AXS Inc.).

[S3] G. M. Sheldrick, *Acta Cryst. A.*, 2008, **64**, 112–122.

[S4] C. F. Macrae, I. Sovago, S. J. Cottrell, P. T. A. Galek, P. McCabe, E. Pidcock, M. Platings, G. P. Shields, J. S. Stevens, M. Towler and P. A. Wood, *J. Appl. Cryst.*, 2020, **53**, 226-235.

[S5] Bielecki, A., Burum,D. P., *J. Magn. Reson. A* **1995**, 116, 215−220.

[S6] Spackman, P. R., Turner, M. J., McKinnon, J. J., Wolff, S. K., Grimwood, D. J., Jayatilaka, D. & Spackman, M. A. (2021). *J. Appl. Cryst.* 54, **3**, 1006–1011.

[S7] Mackenzie, C. F.; Spackman, P. R.; Jayatilaka, D.; Spackman, M. A. CrystalExplorer Model Energies and Energy Frameworks: Extension to Metal Coordination Compounds, Organic Salts, Solvates and Open-Shell Systems. *IUCrJ* **2017**, *4*, 575–587.

[S8] Gaussian 09, Revision D.01, M. J. Frisch, G. W. Trucks, H. B. Schlegel, G. E. Scuseria, M. A. Robb, J. R. Cheeseman, G. Scalmani, V. Barone, B. Mennucci, G. A. Petersson, H. Nakatsuji, M. Caricato, X. Li, H. P. Hratchian, A. F. Izmaylov, J. Bloino, G. Zheng, J. L. Sonnenberg, M. Hada, M. Ehara, K. Toyota, R. Fukuda, J. Hasegawa, M. Ishida, T. Nakajima, Y. Honda, O. Kitao, H. Nakai, T. Vreven, J. A. Montgomery, Jr., J. E. Peralta, F. Ogliaro, M. Bearpark, J. J. Heyd, E. Brothers, K. N. Kudin, V. N. Staroverov, T. Keith, R. Kobayashi, J. Normand, K. Raghavachari, A. Rendell, J. C. Burant, S. S. Iyengar, J. Tomasi, M. Cossi, N. Rega, J. M. Millam, M. Klene, J. E. Knox, J. B. Cross, V. Bakken, C. Adamo, J. Jaramillo, R. Gomperts, R. E. Stratmann, O. Yazyev, A. J. Austin, R. Cammi, C. Pomelli, J. W. Ochterski, R. L. Martin, K. Morokuma, V. G. Zakrzewski, G. A. Voth, P. Salvador, J. J. Dannenberg, S. Dapprich, A. D. Daniels, O. Farkas, J. B. Foresman, J. V. Ortiz, J. Cioslowski, and D. J. Fox, Gaussian, Inc., Wallingford CT, 2013.

[S9] GaussView, Version 5, R. Dennington, T. Keith, and J. Millam, Semichem Inc., Shawnee Mission, KS, 2009.

[S10] Zhao, Y.; Truhlar, D. G. The M06 Suite of Density Functionals for Main Group Thermochemistry, Thermochemical Kinetics, Noncovalent Interactions, Excited States, and Transition Elements: Two New Functionals and Systematic Testing of Four M06-Class Functionals and 12 Other Functionals. *Theor. Chem. Acc.* **2008**, *120* (1), 215–241.

[S11] Lu, T., & Chen, F. (2012). *Multiwfn: A multifunctional wavefunction analyzer.* Journal of Computational Chemistry, 33(5), 580–592.
